# Multivariate Modeling of Some Datasets in Continuous Space and Discrete Time

**DOI:** 10.3390/e27080837

**Published:** 2025-08-06

**Authors:** Rigele Te, Juan Du

**Affiliations:** Department of Statistics, Kansas State University, Manhattan, KS 66506, USA; rigele@ksu.edu

**Keywords:** ARMA margin, covariance matrix functions, Gaussian random field, multivariate spatial process, spatio-temporal model

## Abstract

Multivariate space–time datasets are often collected at discrete, regularly monitored time intervals and are typically treated as components of time series in environmental science and other applied fields. To effectively characterize such data in geostatistical frameworks, valid and practical covariance models are essential. In this work, we propose several classes of multivariate spatio-temporal covariance matrix functions to model underlying stochastic processes whose discrete temporal margins correspond to well-known autoregressive and moving average (ARMA) models. We derive sufficient and/or necessary conditions under which these functions yield valid covariance matrices. By leveraging established methodologies from time series analysis and spatial statistics, the proposed models are straightforward to identify and fit in practice. Finally, we demonstrate the utility of these multivariate covariance functions through an application to Kansas weather data, using co-kriging for prediction and comparing the results to those obtained from traditional spatio-temporal models.

## 1. Introduction

Multivariate space–time datasets frequently arise in environmental science, meteorology, geophysics, and many other fields. Examples include studying the impact of soil greenhouse gas fluxes on global warming potential, or analyzing temperature–precipitation relationships under climate change (see [1,2,3], among others). Typically, temporal data are collected at regularly spaced intervals, in contrast to spatial data that are often recorded at irregular locations, such as weather stations. With the increasing availability and complexity of such datasets, it is essential to develop efficient models that capture their intricate dependence structures.

This paper focuses on constructing valid covariance matrix functions that jointly incorporate spatial and temporal information for multivariate random fields. While the spatial statistics literature includes various spatial models, few account for discrete-time dependencies, despite time series playing a crucial role in most environmental and geophysical processes. Traditional approaches often rely on separable space–time covariance structures, which assume the overall covariance is the product of purely spatial and purely temporal components. While computationally convenient, these models ignore space–time interactions that are often fundamental to the underlying physical processes. An increasing body of work has highlighted the importance of nonseparable models. For example, ref. [4] introduced nonseparable stationary spatio-temporal covariance functions, and subsequent generalizations for both stationary and nonstationary processes were developed in [5,6,7], among others. Applications to environmental data such as air pollution are explored by [8,9], while ref. [10] incorporates an inflated gamma distribution to model precipitation trends with zero inflation. However, most of these models are constructed under the assumption of continuous time. In practice, time is typically observed on a discrete, regular grid, whereas spatial locations are distributed more irregularly. Although some models incorporate discrete time through stochastic differential equations or spectral methods (e.g., [11,12]), these approaches often lack closed-form expressions for the covariance structure. While ref. [13] deals with the univariate case, in this work, we derive explicit covariance matrix functions for multivariate space–time processes with discrete temporal components, where the temporal margins follow well-established autoregressive and moving average (ARMA) models. Leveraging the rich theoretical foundation of ARMA processes along with classical spatial modeling, we aim to build flexible, interpretable, and computationally feasible models.

In many modern scientific applications, such as geosciences, environmental monitoring, and economics, large numbers of variables are observed simultaneously. These variables are often correlated, and borrowing information from related (secondary) variables can improve the prediction of a primary variable, especially when the latter is sparsely observed. For simplicity, spatial variables are often modeled separately, ignoring cross-variable dependencies.A key contribution of this work is the development of multivariate spatial covariance structures that capture both within-variable spatial dependence and cross-variable covariances, while also incorporating discrete time information. This enables more accurate predictions through co-kriging across a wide range of applications. While previous efforts have been made in this direction, many are limited to purely spatial or continuous-time settings, or they rely on Bayesian frameworks. Notable contributions include [14,15,16,17], among others. For example, multivariate Poisson-lognormal spatial models have improved predictions in traffic safety studies [18], and recent works have established kriging formulas [19] and copula-based models [20] for multivariate spatial data. We aim to integrate parameter interpretability from analytic model expressions into a unified space–time framework to facilitate multivariate fitting and co-kriging.

On a global scale, many spatial datasets are collected using spherical coordinates. Euclidean-based distances and covariance structures can become distorted on the sphere, especially over large distances, making spherical modeling critical in geophysical and atmospheric sciences. Recent advances include constructions of isotropic positive definite functions on spheres [21], covariance functions for stationary and isotropic Gaussian vector fields [22], and isotropic variogram matrix functions expressed through ultraspherical polynomials [23]. Drawing from these approaches, we also extend some of our discrete-time multivariate spatio-temporal models to spherical domains to ensure validity across both Euclidean and spherical spaces.

We aim to develop a flexible multivariate spatio-temporal modeling framework that incorporates discrete-time structure, spatial correlation (in both Euclidean and spherical spaces), and cross-variable dependencies. Specifically, we consider a *p*-variate space–time random field

$$\{Z\left(s,t\right)={\left(Z_{1}\left(s,t\right),\ldots ,Z_{p}\left(s,t\right)\right)}^{\prime },s\in S^{d}\;\mathrm{or}\;R^{d},\;t\in Z\},$$

with covariance matrix function

$$C\left(s_{1},s_{2},t_{1},t_{2}\right)=\left(\begin{array}{ccc} C_{1,1}\left(s_{1},s_{2},t_{1},t_{2}\right) & \ldots & C_{1,p}\left(s_{1},s_{2},t_{1},t_{2}\right)\\ \vdots & \ddots & \vdots \\ C_{p,1}\left(s_{1},s_{2},t_{1},t_{2}\right) & \ldots & C_{p,p}\left(s_{1},s_{2},t_{1},t_{2}\right) \end{array}\right),$$

where each entry

$$C_{i,j}\left(s_{1},s_{2},t_{1},t_{2}\right)=\mathrm{Cov}\left(Z_{i}\left(s_{1},t_{1}\right),Z_{j}\left(s_{2},t_{2}\right)\right),$$

for i,j=1,…,p, where $S^{d}$ and $R^{d}$ denote the *d*-dimensional unit sphere and Euclidean space, respectively. The process is stationary in both space and time if E(Z(s,t)) is constant for all (s,t) and $C(s_{0},s_{0}+s;t_{0},t_{0}+t)$ depends only on the spatial lag s and temporal lag *t*. We then denote the spatial and temporal margins as $C(s_{1},s_{2},t,t)$ and $C(s,s,t_{1},t_{2})$, respectively, following [24]. In practice, analyzing multivariate space–time data often begins with marginal exploration, applying time series models to study temporal behavior and multivariate spatial analysis to capture cross-variable structure. Given the substantial research advances in both areas, combining their strengths provides a robust foundation for model development, selection, and estimation.

The remainder of this paper is organized as follows. In Section 2, we propose several classes of multivariate spatio-temporal covariance matrix functions, whose discrete-time margins follow ARMA models. We derive necessary and sufficient conditions for these functions to define valid covariance matrices. Section 3 extends the models to incorporate general ARMA margins. In Section 4, we apply our models to Kansas weather data to demonstrate their performance in spatio-temporal prediction compared to traditional methods.

## 2. Moving-Average-Type Temporal Margin

We begin constructing the foundation of our overall framework by examining the covariance structure corresponding to a first-order moving average (MA(1)) model in the discrete temporal margin. It is straightforward to verify that Equation (1) satisfies the defining properties of an MA(1) process at a fixed spatial location. Notably, this structure does not rely on the assumption of temporal stationarity. The main challenge in proving the validity of Equation (1) lies in its nature as a discrete space–time matrix function that varies across different time scales, making it more complex than simply verifying a static covariance matrix. Theorem 8 in [25] offers useful insights that support the proof of the following Theorem 1 (see Appendix A).

**Theorem** **1.** *Let $G_{0}\left(s_{1},s_{2}\right)$ and $G_{1}\left(s_{1},s_{2}\right),s_{1},s_{2}\in D$, $D\subset R^{d}$ or $S^{d},d\geq 1$ be p×p matrix functions, and let $G_{0}\left(s_{1},s_{2}\right)$ be symmetric, i.e., $G_{0}{\left(s_{1},s_{2}\right)}^{\prime }=G_{0}\left(s_{1},s_{2}\right)$. Then, the  p×p function*

(1) $$C\left(s_{1},s_{2};t\right)=\left\{\begin{array}{cc} G_{0}\left(s_{1},s_{2}\right),\; & \;t=0,\\ G_{1}\left(s_{1},s_{2}\right),\; & \;t=1,\\ G_{1}{\left(s_{2},s_{1}\right)}^{\prime },\; & \;t=-1,\\ 0,\; & \;t\neq 0,\pm 1,t\in Z,\;s_{1},s_{2}\in D. \end{array}\right.$$

*is a covariance matrix function on D×Z if and only if the following two conditions are satisfied:*

*(i)*  *$G_{0}\left(s_{1},s_{2}\right)+G_{1}\left(s_{1},s_{2}\right)+G_{1}{\left(s_{2},s_{1}\right)}^{\prime }$ is a covariance matrix function on D,*
*(ii)*  *$G_{0}\left(s_{1},s_{2}\right)-G_{1}\left(s_{1},s_{2}\right)-G_{1}{\left(s_{2},s_{1}\right)}^{\prime }$ is a covariance matrix function on D.*

This theorem reduces the verification of a complex space–time problem to that of a purely spatial covariance model. Building upon the foundational structure developed earlier, we are now prepared to incorporate a broader range of spatial covariance margins to enrich the class of admissible models. Specifically, we integrate the widely used Matérn-type spatial covariance function into our framework and derive the full set of parameter conditions required to ensure validity. In Theorem 2, we begin with a parsimonious Matérn structure in which all smoothness parameters α in M(h∣v,α) are assumed to be equal, as specified in Equation (4) below. Theorem 3 of [14] provides necessary and sufficient conditions under various settings for Equation (4) to define a valid covariance matrix. These results offer important insights that inform the conditions of the theorem and corollary that follows.

**Theorem** **2.** *Let $v=(v_{1},v_{2},\ldots ,v_{p})$, $\alpha =(\alpha_{1},\alpha_{2})$, and $\beta =(\beta_{1},\beta_{2})$ be constant vectors. $v_{k}\geq 0,\;\alpha_{k}\geq 0,\;-1/2\leq \beta_{k}\leq 1/2$, and let $v_{ij}=\left(v_{i}+v_{j}\right)/2$, $D\subset R^{d}$. The sufficient condition for the p×p matrix function*

(2) $$C\left(h;t\right)=\left\{\begin{array}{cc} cM\left(h|v,\alpha_{1}\right)+\left(1-c\right)M\left(h|v,\alpha_{2}\right),\; & \;t=0,\\ cM\left(h|v,\alpha_{1}\right)\beta_{1}+\left(1-c\right)M\left(h|v,\alpha_{2}\right)\beta_{2},\; & \;t=\pm 1,h\in D\\ 0,\; & \;otherwise, \end{array}\right.$$

*to be a correlation matrix function on D×Z is that the constant c satisfies*

(3) 0≤c≤1.

*And if p≤2, (3) is also necessary.*
*where*

(4) $$M\left(h|v,\alpha \right)=({\left(\rho_{ij}m\left(h|v_{ij},\alpha \right)\right)}_{1\leq i,j\leq p},$$

$m\left(h|v_{ij},\alpha \right)=\frac{2^{1-v_{ij}}}{\Gamma (v_{ij})}{\left(\alpha h\right)}^{v_{ij}}K_{v_{ij}}\left(\alpha h\right)$, i,j=1,2, $\rho_{ij}=\frac{\Gamma {\left(v_{i}+\frac{d}{2}\right)}^{\frac{1}{2}}}{\Gamma {\left(v_{i}\right)}^{\frac{1}{2}}}\frac{\Gamma {\left(v_{j}+\frac{d}{2}\right)}^{\frac{1}{2}}}{\Gamma {\left(v_{j}\right)}^{\frac{1}{2}}}\frac{\Gamma (v_{ij})}{\Gamma (v_{ij}+\frac{d}{2})}$.

The following theorem generalizes the parsimonious Matérn covariance structure by relaxing the constraint that all smoothness parameters α in M(h∣v,α) must be equal, as in Equation (4). Following [14], we assume that $M(h|v,\alpha ,\rho_{12})$ is a general multivariate Matérn covariance function in Theorem 3. In addition, the choice of *c* is assumed to satisfy the conditions of Theorem 2 in [13], ensuring that the main diagonal elements of the resulting matrix structure are valid univariate correlation functions.

**Theorem** **3.** *Let $v=(v_{1},\;v_{2},\;v_{12})$, $\alpha =(\alpha_{1},\;\alpha_{2},\;\alpha_{12})$, $\alpha^{\prime }=\left(\alpha_{1}^{\prime },\;\alpha_{2}^{\prime },\;\alpha_{12}^{\prime }\right)$, $\beta =(\beta_{1},\;\beta_{2})$ be constant vectors. $v_{k}\geq 0,\;\alpha_{k}\geq 0,\;\alpha_{k}^{\prime }\geq 0,\;-1/2\leq \beta_{k}\leq 1/2$, $D\subset R^{d}$. A sufficient and necessary condition for the p×p matrix function, p≤2*

(5) $$C\left(h;t\right)=\left\{\begin{array}{cc} cM\left(h|v,\alpha ,\rho_{12}\right)+\left(1-c\right)M\left(h|v,\alpha^{\prime },\rho_{12}^{\prime }\right),\; & \;t=0,\\ cM\left(h|v,\alpha ,\rho_{12}\right)\beta_{1}+\left(1-c\right)M\left(h|v,\alpha^{\prime },\rho_{12}^{\prime }\right)\beta_{2},\; & \;t=\pm 1,h\in D\\ 0,\; & \;otherwise, \end{array}\right.$$

*to be a correlation matrix function on D×Z is that the constant c satisfies*

(6) $$\begin{array}{c} \underset{h\geq 0,D(h)>0}{inf}\frac{c^{2}{\left(1\pm 2\beta_{1}\right)}^{2}H\left(h\right)+{\left(1-c\right)}^{2}{\left(1\pm 2\beta_{2}\right)}^{2}\tilde{H}\left(h\right)}{\left(1\pm 2\beta_{1}\right)\left(1\pm 2\beta_{2}\right)D\left(h\right)}\geq c\left(c-1\right). \end{array}$$

*given D(h)≠0. Where*

(7) $$M\left(h|v,\alpha ,\rho_{12}\right)=\left[\begin{array}{cc} m_{11}\left(h|v_{1},\alpha_{1}\right) & \rho_{12}m_{12}\left(h|v_{12},\alpha_{12}\right)\\ \rho_{12}m_{12}\left(h|v_{12},\alpha_{12}\right) & m_{22}\left(h|v_{2},\alpha_{2}\right) \end{array}\right],$$

*$m_{ij}\left(h|v_{k},\alpha_{k}\right)=\frac{2^{1-v_{k}}}{\Gamma (v_{k})}{\left(\alpha_{k}h\right)}^{v_{k}}K_{v_{k}}\left(\alpha_{k}h\right)$, i,j=1,2, k=1,2,12.*

$$\begin{array}{c} H\left(h\right)=\frac{\alpha_{1}^{2v_{1}}\alpha_{2}^{2v_{2}}c_{v_{1}}c_{v_{2}}}{{\left(\alpha_{1}^{2}+h^{2}\right)}^{v_{1}+d/2}{\left(\alpha_{2}^{2}+h^{2}\right)}^{v_{2}+d/2}}-\frac{\rho_{12}^{2}\alpha_{12}^{4v_{12}}c_{v_{12}}^{2}}{{\left(\alpha_{12}^{2}+h^{2}\right)}^{2v_{12}+d}}, \end{array}$$

*$\tilde{H}\left(h\right)$ is defined like H(h) with $\alpha_{i}$ replaced with $\alpha_{i}^{\prime }$, i=1,2,12 and*

$$\begin{array}{cc} D(h) & =\frac{\alpha_{1}^{2v_{1}}\alpha_{2}^{\prime 2v_{2}}c_{v_{1}}c_{v_{2}}}{{\left(\alpha_{1}^{2}+h^{2}\right)}^{v_{1}+d/2}{\left(\alpha_{2}^{\prime 2}+h^{2}\right)}^{v_{2}+d/2}}+\frac{\alpha_{1}^{\prime 2v_{1}}\alpha_{2}^{2v_{2}}c_{v_{1}}c_{v_{2}}}{{\left(\alpha_{1}^{\prime 2}+h^{2}\right)}^{v_{1}+d/2}{\left(\alpha_{2}^{2}+h^{2}\right)}^{v_{2}+d/2}}\\ \; & -\frac{2\rho_{12}\rho_{12}^{\prime }\alpha_{12}^{2v_{12}}\alpha_{12}^{\prime 2v_{1}2}c_{v_{12}}^{2}}{{\left(\left(\alpha_{12}^{2}+h^{2}\right)\left(\alpha_{12}^{\prime 2}+h^{2}\right)\right)}^{v_{12}+d/2}}. \end{array}$$

If fact, from [14], $M(h|v,\alpha ,\rho_{12})$ is a valid covariance matrix if and only if

(8) $$\begin{array}{cc} \rho_{12}^{2}\leq & \frac{c_{v1}c_{v2}}{c_{v_{12}}^{2}}\frac{\alpha_{1}^{2v_{1}}\alpha_{2}^{2v_{2}}}{\alpha_{12}^{4v_{12}}}\underset{h\geq 0}{inf}\frac{{\left(\alpha_{12}^{2}+h^{2}\right)}^{2v_{12}+d}}{{\left(\alpha_{1}^{2}+h^{2}\right)}^{v_{1}+d/2}{\left(\alpha_{2}^{2}+t^{2}\right)}^{v_{2}+d/2}}, \end{array}$$

(9) $$\begin{array}{cc} \rho_{12}^{\prime 2}\leq & \frac{c_{v1}c_{v2}}{c_{v_{12}}^{2}}\frac{\alpha_{1}^{\prime 2v_{1}}\alpha_{2}^{\prime 2v_{2}}}{\alpha_{12}^{\prime 4v_{12}}}\underset{h\geq 0}{inf}\frac{{\left(\alpha_{12}^{\prime 2}+h^{2}\right)}^{2v_{12}+d}}{{\left(\alpha_{1}^{\prime 2}+h^{2}\right)}^{v_{1}+d/2}{\left(\alpha_{2}^{\prime 2}+t^{2}\right)}^{v_{2}+d/2}}. \end{array}$$

where $c_{v}=\pi^{-d/2}\Gamma \left(v+d/2\right)/\Gamma \left(v\right)$. Therefore, we can show that H(h)≥0, $\tilde{H}\left(h\right)\geq 0$, and D(h)≥0. Under certain conditions, the minimum of the left-hand side of inequality (8) can be equal to zero, which leads to the following corollary.

**Corollary** **1.** *The sufficient and necessary condition for Equation (5) to be a correlation matrix function can be reduced to 0≤c≤1 in the following cases:*

*(a) When $\alpha_{12}\leq min\left(\alpha_{1},\alpha_{2}\right)$, $\alpha_{12}^{\prime }\leq min\left(\alpha_{1}^{\prime },\alpha_{2}^{\prime }\right)$, $v_{12}=\frac{v_{1}+v_{2}}{2}$,*

$$\begin{array}{c} \rho_{12}^{2}=\frac{c_{v_{1}}c_{v_{2}}}{c_{v_{12}}^{2}}{\left(\frac{\alpha_{12}^{2}}{\alpha_{1}\alpha_{2}}\right)}^{d},\rho_{12}^{\prime 2}=\frac{c_{v_{1}}c_{v_{2}}}{c_{v_{12}}^{2}}{\left(\frac{\alpha_{12}^{\prime 2}}{\alpha_{1}^{\prime }\alpha_{2}^{\prime }}\right)}^{d}. \end{array}$$

*(b) When $\alpha_{12}\geq max\left(\alpha_{1},\alpha_{2}\right)$, $\alpha_{12}^{\prime }\geq max\left(\alpha_{1}^{\prime },\alpha_{2}^{\prime }\right)$, $v_{12}=\frac{v_{1}+v_{2}}{2}$,* 

$$\begin{array}{c} \rho_{12}^{2}=\frac{c_{v_{1}}c_{v_{2}}}{c_{v_{12}}^{2}}{\left(\frac{\alpha_{1}}{\alpha_{12}}\right)}^{2v_{1}}{\left(\frac{\alpha_{2}}{\alpha_{12}}\right)}^{2v_{2}},\rho_{12}^{\prime 2}=\frac{c_{v_{1}}c_{v_{2}}}{c_{v_{12}}^{2}}{\left(\frac{\alpha_{1}^{\prime }}{\alpha_{12}^{\prime }}\right)}^{2v_{1}}{\left(\frac{\alpha_{2}^{\prime }}{\alpha_{12}^{\prime }}\right)}^{2v_{2}}. \end{array}$$

The proofs of the theorems and corollary are deferred to the Appendix A. It is well known that setting v=1/2 in the Matérn covariance function yields the exponential form. This leads to the following example:

**Example** **1.** Let α, $\alpha^{\prime }$, $\rho_{12}$, $\rho_{12}^{\prime }$ and $\beta_{k}\left(k=1,2\right)$ be assumed as in Theorem 3, and take $v_{1}=v_{2}=v_{12}=\frac{1}{2}$; then, A sufficient and necessary condition for the matrix function of exponential type

(10) $$C\left(h;t\right)=\left\{\begin{array}{cc} cE\left(h|\alpha ,\rho_{12}\right)+\left(1-c\right)E\left(h|\alpha^{\prime },\rho_{12}^{\prime }\right),\; & \;t=0,\\ cE\left(h|\alpha ,\rho_{12}\right)\beta_{1}+\left(1-c\right)E\left(h|\alpha^{\prime },\rho_{12}^{\prime }\right)\beta_{2},\; & \;t=\pm 1,h\in D\\ 0,\; & \;otherwise, \end{array}\right.$$

to be a stationary correlation matrix function on D×Z is that the constant *c* satisfies inequality (6). Where

(11) $$E\left(h|\alpha ,\rho_{12}\right)=\left[\begin{array}{cc} e_{11}\left(h|\alpha_{1}\right) & \rho_{12}e_{12}\left(h|\alpha_{12}\right)\\ \rho_{12}e_{12}\left(h|\alpha_{12}\right) & e_{22}\left(h|\alpha_{2}\right) \end{array}\right].$$

$e_{ij}\left(h|\alpha_{k}\right)=exp\left(-\alpha_{k}h\right)$, i,j=1,2, k=1,2,12.

## 3. ARMA Type Temporal Margin

In the previous section, we considered the spatio-temporal covariance structure with a moving average of order one (MA(1)) as the temporal margin. in this section, we extend the covariance matrix to more general cases involving some other autoregressive and moving average (ARMA) temporal margins.

The following model establishes the necessary and sufficient conditions for a valid spatio-temporal covariance matrix with ARMA-type temporal dependence. As before, this theorem assumes uniform α in M(h∣v,α).

**Theorem** **4.** *Let $v=(v_{1},v_{2},v_{12})$, $\beta =(\beta_{1},\beta_{2})$, be constant vectors. $v_{k}\geq 0,\;\alpha_{k}\geq 0,\;-1\leq \beta_{k}\leq 1$, and let $v_{12}=\left(v_{1}+v_{2}\right)/2$, $D\subset R^{d}$ or $S^{d}$. A sufficient condition for the p×p matrix function*

(12) $$C\left(h;t\right)=cM\left(h|v,\alpha_{1}\right)\beta_{1}^{\left|t\right|}+\left(1-c\right)M\left(h|v,\alpha_{2}\right)\beta_{2}^{\left|t\right|},\;t\in Z,h\in D$$

*to be a correlation matrix function on D×Z is that the constant c satisfies the following:*

(13) 0≤c≤1.

*And if p≤2, (13) is also necessary.*
*where*

(14) $$M\left(h|v,\alpha \right)=({\left(\rho_{ij}m\left(h|v_{ij},\alpha \right)\right)}_{1\leq i,j\leq p},$$

*$m\left(h|v_{k},\alpha \right)=\frac{2^{1-v_{k}}}{\Gamma (v_{k})}{\left(\alpha h\right)}^{v_{k}}K_{v_{k}}\left(\alpha h\right)$, i,j=1,2, k=1,2,12, $\rho_{12}=\frac{\Gamma {\left(v_{1}+\frac{d}{2}\right)}^{\frac{1}{2}}}{\Gamma {\left(v_{1}\right)}^{\frac{1}{2}}}\frac{\Gamma {\left(v_{2}+\frac{d}{2}\right)}^{\frac{1}{2}}}{\Gamma {\left(v_{2}\right)}^{\frac{1}{2}}}\frac{\Gamma (v_{12})}{\Gamma (v_{12}+\frac{d}{2})}$.*

We now extend this theorem to various values of α in M(h∣v,α). As in the preceding section, we follow [14] and assume that both $M(h|v,\alpha ,\rho_{12})$ and $M(h|v,\alpha^{\prime })$ below are general multivariate Matérn covariance functions. Furthermore, we assume that the choice of *c* satisfies the conditions in Theorem 4 in [13], ensuring that the main diagonal elements in the resulting matrix structure are valid univariate correlation functions.

**Theorem** **5.** *Let $v=(v_{1},v_{2},v_{12})$, $\alpha =(\alpha_{1},\alpha_{2},\alpha_{12})$, $\alpha^{\prime }=\left(\alpha_{1}^{\prime },\alpha_{2}^{\prime },\alpha_{12}^{\prime }\right)$, $\beta =(\beta_{1},\beta_{2})$ be constant vectors. $v_{k}\geq 0,\;\alpha_{k}\geq 0,\;\alpha_{k}^{\prime }\geq 0,\;-1\leq \beta_{k}\leq 1$, $D\subset R^{d}$ or $S^{d}$. A sufficient and necessary condition for p×p matrix function, p≤2*

(15) $$C\left(h;t\right)=cM\left(h|v,\alpha ,\rho_{12}\right)\beta_{1}^{\left|t\right|}+\left(1-c\right)M\left(h|v,\alpha^{\prime },\rho_{12}^{\prime }\right)\beta_{2}^{\left|t\right|},\;t\in Z,h\in D$$

*to be a correlation matrix function on D×Z is that the constant c satisfies*

(16) $$\begin{array}{c} \underset{h\geq 0,D(h)>0}{inf}\frac{c^{2}{\left(\beta_{1}^{*}\right)}^{2}H\left(h\right)+{\left(1-c\right)}^{2}{\left(\beta_{2}^{*}\right)}^{2}\tilde{H}\left(h\right)}{\left(\beta_{1}^{*}\right)\left(\beta_{2}^{*}\right)D\left(h\right)}\geq c\left(c-1\right). \end{array}$$

*where*

$$M\left(h|v,\alpha ,\rho_{12}\right)=\left[\begin{array}{cc} m_{11}\left(h|v_{1},\alpha_{1}\right) & \rho_{12}m_{12}\left(h|v_{12},\alpha_{12}\right)\\ \rho_{12}m_{12}\left(h|v_{12},\alpha_{12}\right) & m_{22}\left(h|v_{2},\alpha_{2}\right) \end{array}\right],$$

*$m_{ij}\left(h|v_{k},\alpha_{k}\right)=\frac{2^{1-v_{k}}}{\Gamma (v_{k})}{\left(\alpha_{k}h\right)}^{v_{k}}K_{v_{k}}\left(\alpha_{k}h\right)$, $\beta_{i}^{*}=\frac{1-\beta_{i}^{2}}{1+\beta_{i}^{2}-2\beta_{i}cos\left(\omega \right)},i,j=1,2,k=1,2,12$*

$$\begin{array}{c} H\left(h\right)=\frac{\alpha_{1}^{2v_{1}}\alpha_{2}^{2v_{2}}c_{v_{1}}c_{v_{2}}}{{\left(\alpha_{1}^{2}+h^{2}\right)}^{v_{1}+d/2}{\left(\alpha_{2}^{2}+h^{2}\right)}^{v_{2}+d/2}}-\frac{\rho_{12}^{2}\alpha_{12}^{4v_{12}}c_{v_{12}}^{2}}{{\left(\alpha_{12}^{2}+h^{2}\right)}^{2v_{12}+d}}, \end{array}$$

*$\tilde{H}\left(h\right)$ is defined like H(h), with $\alpha_{i}$ replaced with $\alpha_{i}^{\prime }$, i=1,2,12.*

$$\begin{array}{cc} D(h) & =\frac{\alpha_{1}^{2v_{1}}\alpha_{2}^{\prime 2v_{2}}c_{v_{1}}c_{v_{2}}}{{\left(\alpha_{1}^{2}+h^{2}\right)}^{v_{1}+d/2}{\left(\alpha_{2}^{\prime 2}+h^{2}\right)}^{v_{2}+d/2}}+\frac{\alpha_{1}^{\prime 2v_{1}}\alpha_{2}^{2v_{2}}c_{v_{1}}c_{v_{2}}}{{\left(\alpha_{1}^{\prime 2}+h^{2}\right)}^{v_{1}+d/2}{\left(\alpha_{2}^{2}+h^{2}\right)}^{v_{2}+d/2}}\\ \; & -\frac{2\rho_{12}\rho_{12}^{\prime }\alpha_{12}^{2v_{12}}\alpha_{12}^{\prime 2v_{1}2}c_{v_{12}}^{2}}{{\left(\left(\alpha_{12}^{2}+h^{2}\right)\left(\alpha_{12}^{\prime 2}+h^{2}\right)\right)}^{v_{12}+d/2}}. \end{array}$$

Incorporating different $\alpha_{i}$ values into the model allows for more detailed spatial parameterization, enabling a more precise capture of spatial trends. Once again, the condition in this theorem can be simplified under several special cases:

**Corollary** **2.** *The sufficient and necessary condition for Equation (15) to be a correlation matrix function can be reduced to 0≤c≤1 in the following cases:*

*(a) When $\alpha_{12}\leq min\left(\alpha_{1},\alpha_{2}\right)$, $\alpha_{12}^{\prime }\leq min\left(\alpha_{1}^{\prime },\alpha_{2}^{\prime }\right)$, $v_{12}=\frac{v_{1}+v_{2}}{2}$,*

$$\begin{array}{c} \rho_{12}^{2}=\frac{c_{v_{1}}c_{v_{2}}}{c_{v_{12}}^{2}}{\left(\frac{\alpha_{12}^{2}}{\alpha_{1}\alpha_{2}}\right)}^{d},\rho_{12}^{\prime 2}=\frac{c_{v_{1}}c_{v_{2}}}{c_{v_{12}}^{2}}{\left(\frac{\alpha_{12}^{\prime 2}}{\alpha_{1}^{\prime }\alpha_{2}^{\prime }}\right)}^{d}. \end{array}$$

*(b) When $\alpha_{12}\geq max\left(\alpha_{1},\alpha_{2}\right)$, $\alpha_{12}^{\prime }\geq max\left(\alpha_{1}^{\prime },\alpha_{2}^{\prime }\right)$, $v_{12}=\frac{v_{1}+v_{2}}{2}$,* 

$$\begin{array}{c} \rho_{12}^{2}=\frac{c_{v_{1}}c_{v_{2}}}{c_{v_{12}}^{2}}{\left(\frac{\alpha_{1}}{\alpha_{12}}\right)}^{2v_{1}}{\left(\frac{\alpha_{2}}{\alpha_{12}}\right)}^{2v_{2}},\rho_{12}^{\prime 2}=\frac{c_{v_{1}}c_{v_{2}}}{c_{v_{12}}^{2}}{\left(\frac{\alpha_{1}^{\prime }}{\alpha_{12}^{\prime }}\right)}^{2v_{1}}{\left(\frac{\alpha_{2}^{\prime }}{\alpha_{12}^{\prime }}\right)}^{2v_{2}}. \end{array}$$

The proof of this corollary is similar to that of Corollary 1. The temporal margin in both theorems is given by

$$C\left(0,t\right)=cI_{2\times 2}\beta_{1}^{\left|t\right|}+\left(1-c\right)I_{2\times 2}\beta_{2}^{\left|t\right|},\;t\in Z,$$

which is a linear combination of valid correlation matrices. This structure encompasses a family of valid spatio-temporal correlation functions with stationary AR(1) (first-order autoregressive model), AR(2), and ARMA(2,1) temporal margins. The parameters $\alpha_{k}$ and $\nu_{k}$ for k=1,2,12 can be interpreted as the spatial scaling and smoothness parameters, respectively. The parameters $\beta_{1}$ and $\beta_{2}$ govern the temporal dynamics, while *c* serves as a mixing parameter balancing the two components.

To apply the proposed parametric models, one may first use time series techniques to fit ARMA models at each spatial location. This process can help determine the appropriate ARMA order and provide starting values for $\beta_{1}$, $\beta_{2}$, and *c*. Final parameter estimation can then be performed using either maximum likelihood estimation or the weighted least squares method of [26] (see also Equation (22) in [27]). For the spatial component, standard procedures in spatial statistics can be employed to estimate initial values for $\alpha_{i}$ and the cross-correlation parameters ρ, $\rho^{\prime }$. For instance, one can use the fitted parameters from the marginal spatial and cross-correlation functions at different time lags as starting points. Additional insights into the temporal structure can be obtained using tools such as the autocorrelation function (ACF), partial autocorrelation function (PACF), and information criteria like AIC and BIC. Since the temporal margin can initially be analyzed independently, this step provides useful guidance for model selection. Ultimately, the choice of the final model should be guided by space–time fitting criteria, which are generally robust to small variations in the marginal temporal model. Simplicity is also an important consideration in final model selection. Therefore, the proposed models, along with the stepwise estimation strategy, offer a practical and flexible approach by decomposing the complex spatio-temporal modeling problem into two more manageable steps. The proposed framework also provides an intuitive path toward modeling multivariate spatio-temporal processes, where each spatial location may follow an ARMA-type temporal process. One benefit of this approach is that it allows the multivariate MA(1) process to be approximated by analyzing marginal trends. Since the spatial correlation structure can differ across variables and time lags, it is often beneficial to estimate the trend separately at each time lag to obtain more accurate initial values. These components can then be integrated into a unified model, which is subsequently refined using joint estimation.

For the data application presented in the next section, parameter estimation was conducted using the least squares method from [7] and the techniques developed in [27]. Extending these techniques to accommodate general ARMA(*p*, *q*) temporal margins would require further theoretical development of the results presented here. However, such extensions remain computationally feasible, particularly when using Cressie’s weighted least squares approach. We leave the exploration of more complex temporal margins for future research.

## 4. Data Example: Kansas Daily Temperature Data

This dataset is sourced from the National Oceanic and Atmospheric Administration (NOAA) and includes observations from 105 weather stations across Kansas. For our real-data application, we focus on two highly correlated variables: daily maximum and minimum temperatures recorded over 8030 days, from 1 January 1990, to 31 December 2011, across all 105 counties. To preprocess the data, we compute weekly averages over the 8030 days, resulting in 1144 weeks of average maximum and minimum temperatures, which we use as our raw dataset. To reduce short-term variability to obtain a more stable pattern, we compute weekly averages from the daily temperature data. We divide the dataset into training and testing sets: the first 800 weeks (approximately the first fifteen years) are used for training, and the remaining 344 weeks (the last seven years) are used for testing. To detrend and deseasonalize the data, we follow the procedure outlined in [27] by subtracting the overall mean weekly temperature for each calendar week. Specifically:

Let $X_{y,w,i}$ be the weekly average temperature in year *y*, week *w*, location *i*; ${\bar{X}}_{w,i}$ be the average temperature for week *w* at location *i* across *n* years; and $X_{y,w,i}^{*}$ represents the weekly value at location *i* with the seasonal mean removed, defined as:

(17) $$X_{y,w,i}^{*}=X_{y,w,i}-{\bar{X}}_{w,i}$$

where

(18) $${\bar{X}}_{w,i}=\frac{1}{n}\sum_{y=1}^{n}X_{y,w,i},\;w=1,2,\ldots ,52$$

This deseasonalization step removes the dominant annual signal and yields weekly anomalies, which reveals the underlying MA(1) correlation pattern.

**Figure 1:**
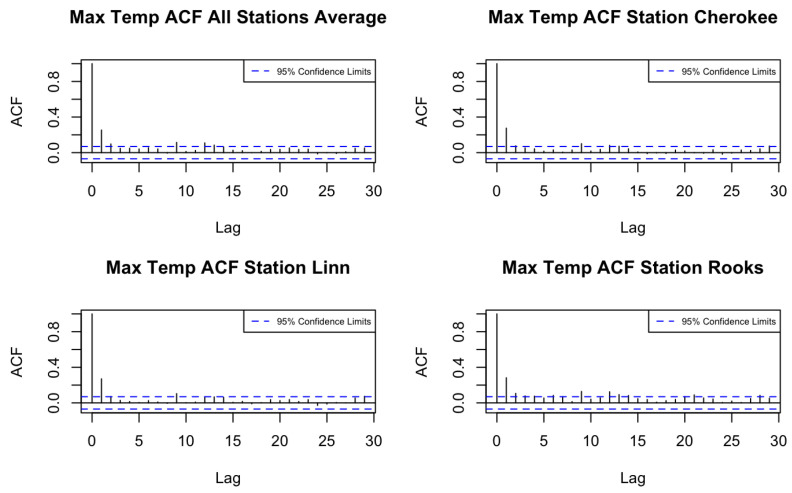
ACFs of maximum temperature in Kansas counties.

**Figure 2:**
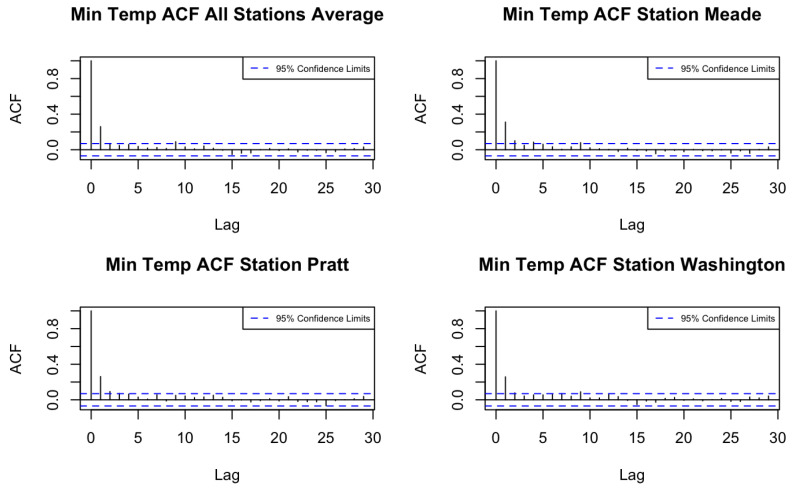
ACFs of minimum temperature in Kansas counties.

We then compute the autocorrelation function (ACF) and cross-correlation function (CCF) of the de-trended minimum and maximum temperature series across the 105 counties using the training period. Figure 1 and Figure 2 display the ACFs of average maximum and minimum temperatures for all locations, as well as for three randomly chosen stations. Based on the ACF and CCF plots, both variables exhibit a pattern consistent with a moving average process of order one (MA(1)), supporting the use of a spatio-temporal model with an MA(1) temporal margin.

The next step is to calculate space–time correlation using detrended data, $X_{y,w,i}$ for model fitting. Since the data includes many location pairs at each distance, it is hard to extract stable spatial trends across time lags. To reduce noise, we apply spatial binning using h=4 and δ=2, which means that we average the spatial correlations within each 4-km bin and discard any empty bins. The binned correlations are the input data for further model fitting. We use the least squares optimization method to fit empirical spatial correlations for minimum temperature, maximum temperature, and their cross-correlation at lag zero. These fits provide suitable initial values for the PMM, SMM, and Cauchy models introduced below.

Guided by this exploratory analysis, using an MA(1)-type temporal margin is a suitable choice for Theorem 3 application. While the correlation approaches 1 at the distance h=0 in Theorem 3, real world data often exhibits a nugget effect and must be accounted for. By incorporating the nugget effect as described in Theorem 3, we formulate the proposed model, referred to as the PMM (Partially Mixed Model) based on C(h;t) in Equation (5), as follows:

(19) $$C_{PMM}\left(h;t\right)=\left[\begin{array}{cc} \left(1-\eta_{1}\right) & 1\\ 1 & \left(1-\eta_{2}\right) \end{array}\right]\circ C\left(h;t\right)+C\left(0;t\right)\circ \left[\begin{array}{cc} \eta_{1}1_{\left\{h=0\right\}} & 0\\ 0 & \eta_{2}1_{\left\{h=0\right\}} \end{array}\right].$$

Cressie’s weighted-least-squares optimization method [26] for parameter estimation (Algorithm 1):
**Algorithm 1** Estimation ProcedureInitialize parameters:   $\theta^{\left(0\right)}=\left(\eta_{1},\eta_{2},\alpha_{11},\alpha_{1},\alpha_{1}^{\prime },\alpha_{2},\alpha_{2}^{\prime }\alpha_{12},\alpha_{12^{\prime }},c,\beta_{1},\beta_{2},\rho_{12},\rho_{12^{\prime }}\right)$;   Set iteration counter d=0;**Repeat**   Compute predicted covariances in Equation (19) at t=0,1,2 across all distances;   Calculate weighted sum of squares:      ${\mathrm{WSS}}^{\left(d\right)}=\sum {\left[\mathrm{residuals}\;\mathrm{at}\;t=0,1,2\;\mathrm{across}\;\mathrm{all}\;\mathrm{distances}\right]}^{2}$;   Update parameters $\theta^{\left(d+1\right)}$ by minimizing ${\mathrm{WSS}}^{\left(d\right)}$ using the L-BFGS-B algorithm;   d←d+1;**until** convergence: $|{\mathrm{WSS}}^{\left(d+1\right)}-{\mathrm{WSS}}^{\left(d\right)}|<\delta$, for a small threshold δ>0.

Finally, the fitted and estimated parameter values for the PMM model are as follows: $\eta_{1}=0.1014$, $\eta_{2}=0.1280$, $\alpha_{1}=0.000025$, $\alpha_{1}^{\prime }=0.004088$, $\alpha_{2}=0.003852$, $\alpha_{2}^{\prime }=0.000025$, $\alpha_{12}=0.002868$, $\alpha_{12}^{\prime }=0.000100$, c=0.5254, $\beta_{1}=0.2496$, $\beta_{2}=0.2591$, $\rho_{12}=0.6964$, $\rho_{12}^{\prime }=0.6523$, and all $v_{ij}$ are set to 2.5. All of the estimated parameters satisfy the conditions in Theorem 3 to ensure Equation (19) is valid as a covariance matrix function. Otherwise, the involved matrix is not invertible, and co-kriging cannot be performed. Next, we apply the purely spatial multivariate Matérn model (SMM), as proposed in [14], for comparison with the incorporated nugget effect.

(20) $$C\left(h\right)=\left[\begin{array}{cc} \left(1-\eta_{1}\right) & 1\\ 1 & \left(1-\eta_{2}\right) \end{array}\right]\circ \left\{M(h|v,\alpha ,\rho_{12})\right\}+\left[\begin{array}{cc} \eta_{1}1_{\left\{h=0\right\}} & 0\\ 0 & \eta_{2}1_{\left\{h=0\right\}} \end{array}\right].$$

In addition, we compared the performance of the Cauchy separable model in continuous time, as proposed in [14], with the nugget effect incorporated.

(21) $$C\left(h;t\right)=\left\{\left[\begin{array}{cc} \left(1-\eta_{1}\right) & 1\\ 1 & \left(1-\eta_{2}\right) \end{array}\right]\circ M\left(h|v,\alpha ,\rho_{12}\right)+\left[\begin{array}{cc} \eta_{1}1_{h=0} & 0\\ 0 & \eta_{2}1_{h=0} \end{array}\right]\right\}\cdot \{(1+{a|t|}^{2\alpha }{)}^{-1}\},$$

where t∈R,h∈D.

**Figure 3:**
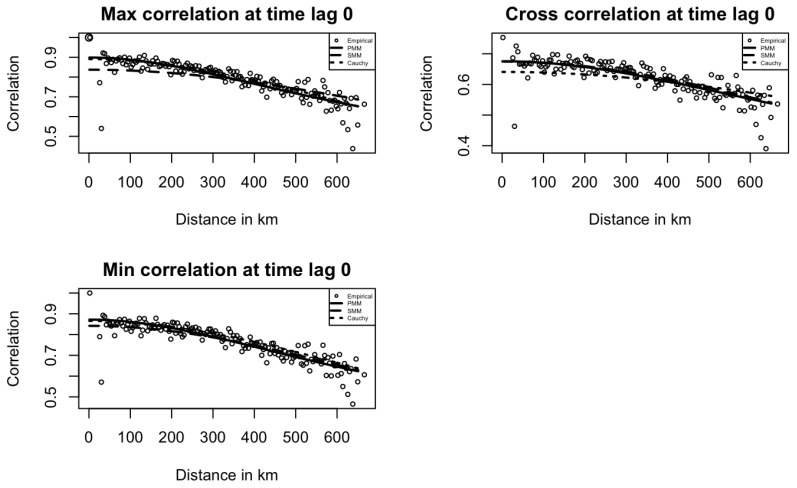
Empirical Temperature space–time correlations and fitted models at time lag 0 in Kansas.

**Figure 4:**
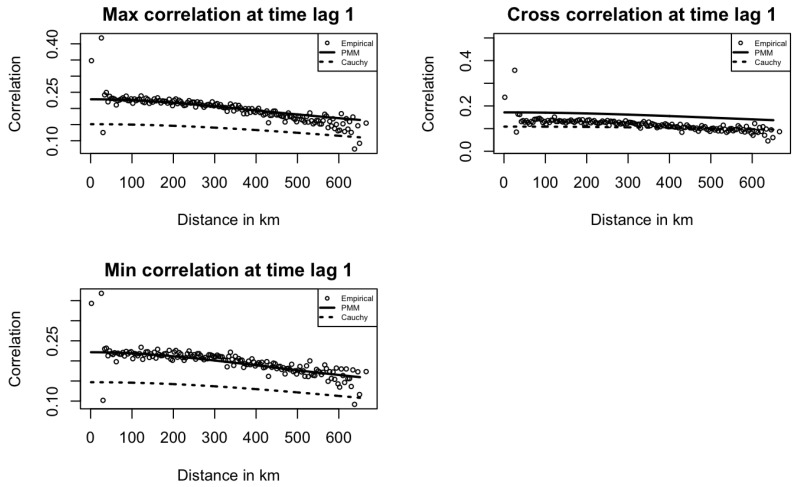
Empirical Temperature space–time correlations and fitted models at time lag 1 in Kansas.

Figure 3 and Figure 4 show the fitted PMM, SMM, and Cauchy models at time lags of 0 and 1 for maximum temperature, minimum temperature, and their cross space–time correlations. In Figure 3, the PMM model fits the empirical correlations better than the SMM and Cauchy models, capturing the underlying structure more accurately. In Figure 4, for maximum and minimum temperature correlations at lag 1, the PMM better capture the correlation patterns, while the Cauchy model performs slightly better for the cross-correlation.

**Figure 5:**
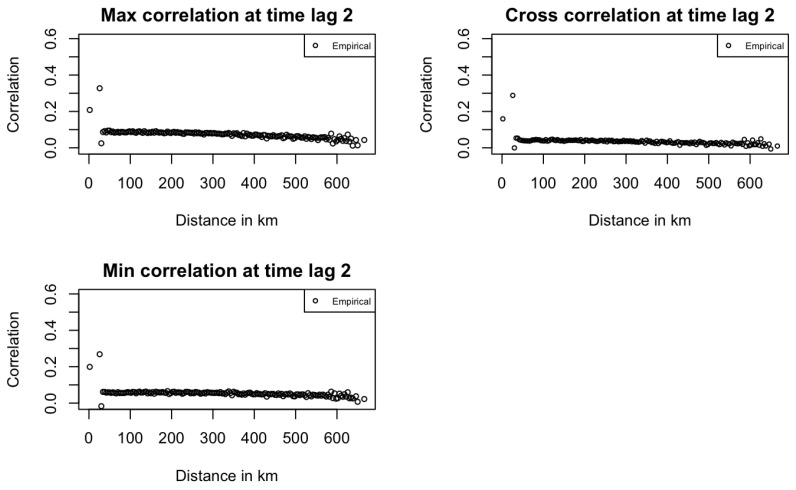
Empirical Temperature space–time correlations at time lag 2 in Kansas.

Across both figures, correlation dispersion increases at long distances, as seen in the first plot of Figure 3. This pattern aligns with real-world expectations, where correlation typically decreases with distance, and it also contributes to reduced model fitting performance. Figure 5 shows that at time lag 2, all correlations are near zero, highlighting the MA(1) temporal structure in the data. PMM model correlation estimates are also zero by definition.

After fitting the PMM, SMM, and Cauchy models on the training data, the next step is to perform co-kriging for prediction on the testing data, as described below.

The response variable $\hat{Y}\left(s_{0},t_{0}\right)$ at location $s_{0}$, time $t_{0}$ is estimated as follows:

(22) $$\hat{Y}\left(s_{0},t_{0}\right)=\sum_{i=1}^{n}\sum_{j=1}^{m}\lambda_{ij}^{Y}Y\left(s_{i},t_{j}\right)+\sum_{i=1}^{n}\sum_{j=1}^{m}\lambda_{ij}^{X}X\left(s_{i},t_{j},\right).$$

where the weights $\lambda_{ij}^{Y}$ and $\lambda_{ij}^{X}$ are obtained by solving the following:

(23) $$\lambda_{\mathrm{ck}}=K_{\mathrm{ck}}^{-1}k_{\mathrm{ck}},$$

(24) $$K_{\mathrm{ck}}=\left(\begin{array}{cccc} C_{YY} & C_{YX} & 1 & 0\\ C_{XY} & C_{XX} & 0 & 1\\ 1^{⊤} & 0^{⊤} & 0 & 0\\ 0^{⊤} & 1^{⊤} & 0 & 0 \end{array}\right),$$

where:

(25) $$C_{YY}=\left(\begin{array}{ccc} C_{YY}\left(s_{i}-s_{j},t_{1}-t_{1}\right) & \ldots & C_{YY}\left(s_{i}-s_{j},t_{1}-t_{m}\right)\\ \vdots & \ddots & \vdots \\ C_{YY}\left(s_{i}-s_{j},t_{m}-t_{1}\right) & \ldots & C_{YY}\left(s_{i}-s_{j},t_{m}-t_{m}\right) \end{array}\right).$$

$C_{YY}\left(s_{i}-s_{j},t_{1}-t_{1}\right)$ is the covariance matrix across distances at time lag $t_{1}-t_{1}$ for variable *Y* and

(26) $$k_{\mathrm{ck}}=\left(\begin{array}{c} C_{YY}\left(s_{0}-s_{1},t_{0}-t_{1}\right)\\ \vdots \\ C_{YY}\left(s_{0}-s_{n},t_{0}-t_{m}\right)\\ C_{YX}\left(s_{0}-s_{1},t_{0}-t_{1}\right)\\ \vdots \\ C_{YX}\left(s_{0}-s_{n},t_{0}-t_{m}\right)\\ 1\\ 0 \end{array}\right).$$

In the PMM and Cauchy models, co-kriging is performed using the minimum and maximum values across all locations at t−1 and t−2 as input data, and SMM model uses t−1 only.

In addition, we consider a traditional time series modeling approach. Since standard time series prediction functions in the R package do not support forecasting with fixed parameters, we developed a custom implementation using the Innovations algorithm described in [4]. We fit the time series model on the training data for maximum temperatures at all 105 stations. Specifically, for each station, we estimated the parameters θ and σ, which are the key components of a moving average process of order one (MA(1)), and we used them to generate predictions on the testing data.

Finally, predictions were obtained for the testing period. The root mean squared error (RMSE) and 95% data interval was computed for each method to assess predictive performance. Table 1 reports the model performance across all counties for maximum temperature.

The percentage of Stations with the Lowest RMSE shows that the PMM model outperforms the others at most locations, achieving the lowest RMSE at 93.3% of the 105 stations. This demonstrates the model’s broad applicability and consistency across different locations. Consequently, the PMM model also produces the lowest average RMSE across all locations. While this difference may seem small, it is important in the de-seasonalized weekly average temperature data, where fluctuations are limited, making even small improvements both statistically and practically meaningful; see [28]. Moreover, the PMM model proves to be more reliable at individual stations, consistently providing better local predictions. This suggests that the model captures more complex spatial structures than simple MA(1) temporal margin or simple spatial correlation margin like the SMM model. The models used for comparison also have strong performance, using the marginal average as the starting points. All models shared those initial value together, so slight improvements are still considered beneficial. Based on this analysis, the proposed PMM model demonstrates consistently better predictive performance, particularly when the temporal margin of the space–time process is properly modeled using an MA(1) structure. The PMM model can also perform well when there is a large number of missing values in the primary variable by leveraging information from the secondary variable, which time series models are unable to utilize. Also, in real-world applications involving complex spatial–temporal data, model selection can be challenging. The PMM model stands out as an easy choice by simply using the marginal spatial and temporal correlations to select the appropriate structure in the PMM model. These results suggest that incorporating both strongly correlated spatial components and discrete-time dependence improves the overall predictive accuracy.

## 5. Discussion

This work presents a foundational framework for direct modeling of space–time random fields with spatially correlated structures and time series components. The methodology developed here enables the integration of spatial covariance models with some autoregressive and moving average temporal structures, offering a tractable yet flexible approach for analyzing spatio-temporal data. Looking ahead, several avenues for further development are promising. One direction is to incorporate more complex forms of temporal dependence, such as general ARMA or nonstationary time dynamics, to better reflect the intricate temporal behaviors observed in environmental and geophysical data. From an inferential standpoint, parameter estimation techniques can be enhanced by moving beyond least squares approaches. Specifically, adopting maximum likelihood estimation to fit the full correlation structure could lead to more efficient and statistically robust inference, particularly when the data exhibits strong space–time interactions. Additionally, while some of the current framework relies on the Matérn class of spatial covariance functions due to its theoretical and practical appeal, other families of spatial structures, including compactly supported or nonstationary models, may offer advantages in specific applications. Exploring these alternatives can further improve the adaptability of the modeling strategy to diverse scientific domains.

## Figures and Tables

**Table 1:** Kansas maximum temperature RMSE statistics.

Measure	PMM	SMM	Cauchy	Time Series
% Stations w/Lowest RMSE	93.3%	4.8%	0%	1.9%
AVG. RMSE at All Stations	3.887092	4.686701	3.938303	3.914282
95% Data Interval	[3.81, 3.96]	[4.64, 4.73]	[3.86, 4.02]	[3.84, 3.99]

## Data Availability

The raw data supporting the conclusions of this paper will be made available by the authors upon request.

## Abbreviations

The following abbreviations are used in this manuscript:

ARMA	Autoregressive and Moving Average
ACF	Autocorrelation Function (ACF)
PMM	Partially Mixed Model
RMSE	Root Mean Squared Error

## Appendix A. Proofs

**Proof of Theorem** **1.** Under the assumptions (i) and (ii), we are going to apply Theorem 8 of [29] to verify that (1) is a covariance matrix function on D×Z. Clearly, ${\left\{C\left(s_{1},s_{2};t\right)\right\}}^{\prime }=C\left(s_{2},s_{1};-t\right),s_{1},s_{2}\in D,t\in Z$. Thus, it suffices that the inequality

$$\sum_{i=1}^{n}\sum_{j=1}^{n}a_{i}^{\prime }C\left(s_{i},s_{j};i-j\right)a_{j}\geq 0$$

or, equivalently,

(A1) $$\sum_{i=1}^{n}a_{i}^{\prime }G_{0}\left(s_{i},s_{i}\right)a_{i}+\sum_{i=1}^{n-1}\left\{a_{i}^{\prime }G_{1}^{\prime }\left(s_{i+1},s_{i}\right)a_{i+1}+a_{i+1}^{\prime }G_{1}\left(s_{i+1},s_{i}\right)a_{i}\right\}\geq 0,$$

holds for every positive integer *n*, any $s_{k}\in D$, and any $a_{k}\in R^{m}$. Since $G_{0}\left(s_{1},s_{2}\right)+G_{1}\left(s_{1},s_{2}\right)+G_{1}^{\prime }\left(s_{1},s_{2}\right)$ is a covariance matrix function on D, its transpose ${\left\{G_{0}\left(s_{1},s_{2}\right)+G_{1}\left(s_{1},s_{2}\right)+G_{1}^{\prime }\left(s_{1},s_{2}\right)\right\}}^{\prime }=G_{0}\left(s_{1},s_{2}\right)+G_{1}^{\prime }\left(s_{1},s_{2}\right)+G_{1}\left(s_{1},s_{2}\right)$ equals $G_{0}\left(s_{2},s_{1}\right)+G_{1}\left(s_{2},s_{1}\right)+G_{1}^{\prime }\left(s_{2},s_{1}\right)$, so that

$$G_{1}^{\prime }\left(s_{1},s_{2}\right)+G_{1}\left(s_{1},s_{2}\right)=G_{1}\left(s_{2},s_{1}\right)+G_{1}^{\prime }\left(s_{2},s_{1}\right),\;\;\;\;\;s_{1},s_{2}\in D.$$

Notice that the matrix function $\frac{1}{2}\left(C\left(s_{1},s_{2};t\right)+C^{\prime }\left(s_{1},s_{2};t\right)\right)$ can be written as follows:

$$\begin{array}{ccc} & & \frac{1}{2}\left(C\left(s_{1},s_{2};t\right)+C^{\prime }\left(s_{1},s_{2};t\right)\right)\\ & = & \left\{\begin{array}{cc} G_{0}\left(s_{1},s_{2}\right),\; & \;t=0,\\ \frac{1}{2}\left\{G_{1}\left(s_{1},s_{2}\right)+G_{1}^{\prime }\left(s_{1},s_{2}\right)\right\},\; & \;t=1,\\ \frac{1}{2}\left\{G_{1}^{\prime }\left(s_{2},s_{1}\right)+G_{1}\left(s_{2},s_{1}\right)\right\},\; & \;t=-1,\\ 0,\; & \;t\pm 2,\pm 3,\ldots ,\;s_{1},s_{2}\in D, \end{array}\right.\\ & = & \left\{\begin{array}{cc} G_{0}\left(s_{1},s_{2}\right),\; & \;t=0,\\ \frac{1}{2}\left\{G_{1}\left(s_{1},s_{2}\right)+G_{1}^{\prime }\left(s_{1},s_{2}\right)\right\},\; & \;t=1,\\ \frac{1}{2}\left\{G_{1}\left(s_{1},s_{2}\right)+G_{1}^{\prime }\left(s_{1},s_{2}\right)\right\},\; & \;t=-1,\\ 0,\; & \;t\pm 2,\pm 3,\ldots ,\;s_{1},s_{2}\in D, \end{array}\right.\\ & = & \frac{G_{0}\left(s_{1},s_{2}\right)+G_{1}\left(s_{1},s_{2}\right)+G_{1}^{\prime }\left(s_{1},s_{2}\right)}{2}\cdot \left\{\begin{array}{cc} I,\; & \;t=0,\\ \frac{1}{2}I,\; & \;t=1,\\ \frac{1}{2}I,\; & \;t=-1,\\ 0,\; & \;t=\pm 2,\pm 3,\ldots ,\;s_{1},s_{2}\in D, \end{array}\right.\\ & & +\frac{G_{0}\left(s_{1},s_{2}\right)-G_{1}\left(s_{1},s_{2}\right)-G_{1}^{\prime }\left(s_{1},s_{2}\right)}{2}\cdot \left\{\begin{array}{cc} I,\; & \;t=0,\\ -\frac{1}{2}I,\; & \;t=1,\\ -\frac{1}{2}I,\; & \;t=-1,\\ 0,\; & \;t=\pm 2,\pm 3,\ldots ,\;s_{1},s_{2}\in D. \end{array}\right. \end{array}$$

This is a sum of two separable covariance matrix functions and is thus a covariance matrix function on D×Z. Based on Theorem 8 of [29], we obtain

$$\begin{array}{ccc} 0 & \leq & \frac{1}{2}\sum_{i=1}^{n}\sum_{j=1}^{n}a_{i}^{\prime }\left\{C\left(s_{i},s_{j};i-j\right)+C^{\prime }\left(s_{i},s_{j};i-j\right)\right\}a_{j}\\ & = & \frac{1}{2}\sum_{i=1}^{n}a_{i}^{\prime }\left\{C\left(s_{i},s_{i};0\right)+C^{\prime }\left(s_{i},s_{i};0\right)\right\}a_{i}\\ & & +\frac{1}{2}\sum_{i=1}^{n-1}a_{i}^{\prime }\left\{C\left(s_{i},s_{i+1};-1\right)+C^{\prime }\left(s_{i},s_{i+1};-1\right)\right\}a_{i+1}\\ & & +\frac{1}{2}\sum_{i=1}^{n-1}a_{i+1}^{\prime }\left\{C\left(s_{i+1},s_{i};1\right)+C^{\prime }\left(s_{i+1},s_{i};1\right)\right\}a_{i}\\ & = & \sum_{i=1}^{n}a_{i}^{\prime }G_{0}\left(s_{i},s_{i}\right)a_{i}+\frac{1}{2}\sum_{i=1}^{n-1}a_{i}^{\prime }\left\{G_{1}^{\prime }\left(s_{i+1},s_{i}\right)+G_{1}\left(s_{i+1},s_{i}\right)\right\}a_{i+1}\\ & & +\frac{1}{2}\sum_{i=1}^{n-1}a_{i+1}^{\prime }\left\{G_{1}\left(s_{i+1},s_{i}\right)+G_{1}^{\prime }\left(s_{i+1},s_{i}\right)\right\}a_{i}\\ & = & \sum_{i=1}^{n}a_{i}^{\prime }G_{0}\left(s_{i},s_{i}\right)a_{i}+\sum_{i=1}^{n-1}\left\{a_{i}^{\prime }G_{1}^{\prime }\left(s_{i+1},s_{i}\right)a_{i+1}+a_{i+1}^{\prime }G_{1}\left(s_{i+1},s_{i}\right)a_{i}\right\}, \end{array}$$

where the last equality follows from $a_{i}^{\prime }G_{1}\left(s_{i+1},s_{i}\right)a_{i+1}=a_{i+1}^{\prime }G_{1}^{\prime }\left(s_{i+1},s_{i}\right)\}a_{i}$. Thus, inequality (A1) is derived. Conversely, suppose that Equation (1) is a covariance matrix function on D×Z. Then, for arbitrary *n* locations and *l* integer time points at each location, we formulate nm pairs $s_{i}$ and $t_{j}=j$, choose the corresponding vectors as the products $a_{i}b_{j}$, i=1,…,n,j=1,…,l, and obtain

$$\sum_{i=1}^{n}\sum_{i^{\prime }=1}^{n}\sum_{j=1}^{l}\sum_{j^{\prime }=1}^{l}b_{j}b_{j^{\prime }}a_{i}^{\prime }C\left(s_{i},s_{i^{\prime }};j-j^{\prime }\right)a_{i^{\prime }}\geq 0,$$

or

$$\sum_{i=1}^{n}\sum_{i^{\prime }=1}^{n}a_{i}^{\prime }\left(\sum_{j=1}^{l}b_{j}^{2}C\left(s_{i},s_{i^{\prime }};0\right)+\sum_{j=1}^{l-1}b_{j}b_{j+1}\left(C\left(s_{i},s_{i^{\prime }};1\right)+C\left(s_{i},s_{i^{\prime }};-1\right)\right)\right)a_{i^{\prime }}\geq 0,$$

or

(A2) $$\sum_{i=1}^{n}\sum_{i^{\prime }=1}^{n}a_{i}^{\prime }\left(\sum_{j=1}^{l}b_{j}^{2}G_{0}\left(s_{i},s_{i^{\prime }}\right)+\sum_{j=1}^{l-1}b_{j}b_{j+1}\left(G_{1}\left(s_{i},s_{i^{\prime }}\right)+G_{1}^{\prime }\left(s_{i^{\prime }},s_{i}\right)\right)\right)a_{i^{\prime }}\geq 0.$$

In particular, in Equation (A2), taking $b_{j}=1,j=1,\ldots ,l$ and dividing both sides by *l* yields

$$\sum_{i=1}^{n}\sum_{i^{\prime }=1}^{n}a_{i}^{\prime }\left(G_{0}\left(s_{i},s_{i^{\prime }}\right)+\frac{l-1}{l}\left(G_{1}\left(s_{i},s_{i^{\prime }}\right)+G_{1}^{\prime }\left(s_{i^{\prime }},s_{i}\right)\right)\right)a_{i^{\prime }}\geq 0.$$

Letting l→∞ gives

$$\sum_{i=1}^{n}\sum_{i^{\prime }=1}^{n}a_{i}^{\prime }\left(G_{0}\left(s_{i},s_{i^{\prime }}\right)+G_{1}\left(s_{i},s_{i^{\prime }}\right)+G_{1}^{\prime }\left(s_{i^{\prime }},s_{i}\right)\right)a_{i^{\prime }}\geq 0.$$

This implies that $G_{0}\left(s_{1},s_{2}\right)+G_{1}\left(s_{1},s_{2}\right)+G_{1}^{\prime }\left(s_{2},s_{1}\right)$ is a covariance matrix function on S, based on Theorem 8 of [29]. Thus, condition (i) is confirmed. Similarly, in order to confirm condition (ii), in Equation (A2), we take $b_{j}={\left(-1\right)}^{j},j=1,\ldots ,l$, divide both sides by *l*, and obtain

$$\sum_{i=1}^{n}\sum_{i^{\prime }=1}^{n}a_{i}^{\prime }\left(G_{0}\left(s_{i},s_{i^{\prime }}\right)-\frac{l-1}{l}\left(G_{1}\left(s_{i},s_{i^{\prime }}\right)+G_{1}^{\prime }\left(s_{i^{\prime }},s_{i}\right)\right)\right)a_{i^{\prime }}\geq 0.$$

Letting l→∞ gives

$$\sum_{i=1}^{n}\sum_{i^{\prime }=1}^{n}a_{i}^{\prime }G_{0}\left(s_{i},s_{i^{\prime }}\right)-G_{1}\left(s_{i},s_{i^{\prime }}\right)-G_{1}^{\prime }\left(s_{i^{\prime }},s_{i}\right))a_{i^{\prime }}\geq 0.$$

This implies that $G_{0}\left(s_{1},s_{2}\right)-G_{1}\left(s_{1},s_{2}\right)-G_{1}^{\prime }\left(s_{2},s_{1}\right)$ is a covariance matrix function on S, based on Theorem 8 of [29]. □

**Proof of Theorem** **2.** Following from Theorem 1, it is equivalent to show that the inequality (3) is a necessary and sufficient condition for $G_{0}\left(h\right)\pm G_{1}\left(h\right)\pm G_{1}{\left(h\right)}^{\prime }$ to be a valid covariance matrix function on D with $h=\left|\right|s_{i}-s_{j}\left|\right|$. Under the scenario of this theorem,

(A3) $$G_{0}\left(h\right)\pm G_{1}\left(h\right)\pm G_{1}{\left(h\right)}^{\prime }=cM\left(h|v,\alpha_{1}\right)\left(1\pm 2\beta_{1}\right)+\left(1-c\right)M\left(h|v,\alpha_{2}\right)\left(1\pm 2\beta_{2}\right),h\in D.$$

For sufficiency in general, by applying Theorem 2 in [22], A positive linear combination of two covariance matrix functions is also a valid covariance matrix function on D. Since condition (3) holds—i.e., 0≤c≤1, $-\frac{1}{2}\leq \beta_{i}\leq \frac{1}{2}$ for i=1,2, and M(h) is a spatial correlation matrix function, Equation (A3) is also a valid covariance matrix function. Furthermore, by Theorem 1, Equation (2) defines a valid spatio-temporal covariance matrix function. When p≤2, we will show condition (3) is both sufficient and necessary. To this end, let’s consider the spectral density of the Matérn class of a function (see Equation (32) of [30]). The Fourier transforms of $G_{0}\left(h\right)+G_{1}\left(h\right)+G_{1}{\left(h\right)}^{\prime }$ and $G_{0}\left(h\right)-G_{1}\left(h\right)-G_{1}{\left(h\right)}^{\prime }$ are given as follows:

$$F\left(h\right)=\left[\begin{array}{cc} c_{\nu_{1}}f_{11}\left(h\right) & c_{\nu_{12}}\rho_{12}f_{12}\left(h\right)\\ c_{\nu_{12}}\rho_{12}f_{21}\left(h\right) & c_{\nu_{2}}f_{22}\left(h\right) \end{array}\right];\;\;\;G\left(h\right)=\left[\begin{array}{cc} c_{\nu_{1}}g_{11}\left(h\right) & c_{\nu_{12}}\rho_{12}g_{12}\left(h\right)\\ c_{\nu_{12}}\rho_{12}g_{21}\left(h\right) & c_{\nu_{2}}g_{22}\left(h\right) \end{array}\right],$$

where $c_{\nu }=\pi^{-d/2}\Gamma \left(\nu +d/2\right)/\Gamma \left(\nu \right)$,

$$\begin{array}{c} f_{ij}\left(h\right)=c{\left(\alpha_{1}\right)}^{v_{i}+v_{j}}{\left(h^{2}+\alpha_{1}^{2}\right)}^{-\frac{v_{i}+v_{j}}{2}-d/2}\left(1+2\beta_{1}\right)+\left(1-c\right)\alpha_{2}^{v_{i}+v_{j}}{\left(h^{2}+\alpha_{2}^{2}\right)}^{-\frac{v_{i}+v_{j}}{2}-d/2}\left(1+2\beta_{2}\right), \end{array}$$

and

$$\begin{array}{c} g_{ij}\left(h\right)=c{\left(\alpha_{1}\right)}^{v_{i}+v_{j}}{\left(h^{2}+\alpha_{1}^{2}\right)}^{-\frac{v_{i}+v_{j}}{2}-d/2}\left(1-2\beta_{1}\right)+\left(1-c\right)\alpha_{2}^{v_{i}+v_{j}}{\left(h^{2}+\alpha_{2}^{2}\right)}^{-\frac{v_{i}+v_{j}}{2}-d/2}\left(1-2\beta_{2}\right)\\ s\in D. \end{array}$$

respectively. Hence, it is reduced to show that inequality (3) is necessary and sufficient for F(h) and G(h) to be nonnegative definite, which means $f_{11}\left(h\right)\geq 0$, $f_{22}\left(h\right)\geq 0$, $g_{11}\left(h\right)\geq 0$, $g_{22}\left(h\right)\geq 0$ and

(A4) $$\begin{array}{cc} c_{v_{1}}c_{v_{2}}f_{11}\left(h\right)f_{22}\left(h\right)-c_{v_{12}}^{2}\rho_{12}^{2}f_{12}\left(h\right)f_{21}\left(h\right) & \geq 0, \end{array}$$

(A5) $$\begin{array}{cc} c_{v_{1}}c_{v_{2}}g_{11}\left(h\right)g_{22}\left(h\right)-c_{v_{12}}^{2}\rho_{12}^{2}g_{12}\left(h\right)g_{21}\left(h\right) & \geq 0, \end{array}$$

based on Cramér’s Theorem [31]. From Theorem 2 in [13], we already know that $f_{11}\left(h\right)\geq 0$ and $g_{11}\left(h\right)\geq 0$ if and only if

(A6) $$\begin{array}{c} {\left\{1-\frac{\alpha_{2}^{d}\left(1-2\beta_{1}\right)}{\alpha_{1}^{d}\left(1-2\beta_{2}\right)}\right\}}^{-1}\leq c\leq {\left\{1-\frac{\alpha_{1}^{2v_{1}}\left(1+2\beta_{1}\right)}{\alpha_{2}^{2v_{1}}\left(1+2\beta_{2}\right)}\right\}}^{-1}. \end{array}$$

Also, $f_{22}\left(h\right)\geq 0$ and $g_{22}\left(h\right)\geq 0$ if and only if

(A7) $$\begin{array}{c} {\left\{1-\frac{\alpha_{2}^{d}\left(1-2\beta_{1}\right)}{\alpha_{1}^{d}\left(1-2\beta_{2}\right)}\right\}}^{-1}\leq c\leq {\left\{1-\frac{\alpha_{1}^{2v_{2}}\left(1+2\beta_{1}\right)}{\alpha_{2}^{2v_{2}}\left(1+2\beta_{2}\right)}\right\}}^{-1}. \end{array}$$

Since $0\leq v_{1}\leq v_{2},0\leq \alpha_{1}\leq \alpha_{2},-1/2\leq \beta_{1}\leq \beta_{2}\leq 1/2$, $f_{ii}\geq 0$ and $g_{ii}\geq 0$, i=1,2, entail

(A8) $$\begin{array}{c} {\left\{1-\frac{\alpha_{2}^{d}\left(1-2\beta_{1}\right)}{\alpha_{1}^{d}\left(1-2\beta_{2}\right)}\right\}}^{-1}\leq c\leq {\left\{1-\frac{\alpha_{1}^{2v_{2}}\left(1+2\beta_{1}\right)}{\alpha_{2}^{2v_{2}}\left(1+2\beta_{2}\right)}\right\}}^{-1}. \end{array}$$

To evaluate inequalities (A4) and (A5), noting that $c_{v_{1}}c_{v_{2}}=c_{v_{12}}^{2}\rho_{12}^{2}$, we expand the LHS of Equations (A4) and (A5) with this positive factor removed.

$$\begin{array}{cc} \; & c_{v_{1}}c_{v_{2}}(c\alpha_{1}^{2v_{1}}{\left(||h|\right|}^{2}+\alpha_{1}^{2}{)}^{-v_{1}-d/2}\left(1\pm 2\beta_{1}\right)+\left(1-c\right)\alpha_{2}^{2v_{1}}{\left(h^{2}+\alpha_{2}^{2}\right)}^{-v_{1}-d/2}\left(1\pm 2\beta_{2}\right))\\ \; & \cdot (c\alpha_{1}^{2v_{2}}{\left(h^{2}+\alpha_{1}^{2}\right)}^{-v_{2}-d/2}\left(1\pm 2\beta_{1}\right)+\left(1-c\right)\alpha_{2}^{2v_{2}}{\left(h^{2}+\alpha_{2}^{2}\right)}^{-v_{2}-d/2}\left(1\pm 2\beta_{2}\right))\\ \; & -c_{v_{12}}^{2}\rho_{12}^{2}{\left(c\alpha_{1}^{v_{1}+v_{2}}{\left(h^{2}+\alpha_{1}^{2}\right)}^{-\frac{v_{1}+v_{2}}{2}-d/2}\left(1\pm 2\beta_{1}\right)+\left(1-c\right)\alpha_{2}^{v_{1}+v_{2}}{\left(h^{2}+\alpha_{2}^{2}\right)}^{-\frac{v_{1}+v_{2}}{2}-d/2}\left(1\pm 2\beta_{2}\right)\right)}^{2}\\ = & \;c^{2}\alpha_{1}^{2v_{1}+2v_{2}}{\left(h^{2}+\alpha_{1}^{2}\right)}^{-v_{1}-d/2}{\left(h^{2}+\alpha_{1}^{2}\right)}^{-v_{2}-d/2}{\left(1\pm 2\beta_{1}\right)}^{2}\\ \; & +c\left(1-c\right)\alpha_{1}^{2v_{2}}\alpha_{2}^{2v_{1}}{\left(h^{2}+\alpha_{1}^{2}\right)}^{-v_{2}-d/2}{\left(h^{2}+\alpha_{2}^{2}\right)}^{-v_{1}-d/2}\left(1\pm 2\beta_{1}\right)\left(1\pm 2\beta_{2}\right)\\ \; & +c\left(1-c\right)\alpha_{1}^{2v_{1}}\alpha_{2}^{2v_{2}}{\left(h^{2}+\alpha_{1}^{2}\right)}^{-v_{1}-d/2}{\left(h^{2}+\alpha_{2}^{2}\right)}^{-v_{2}-d/2}\left(1\pm 2\beta_{1}\right)\left(1\pm 2\beta_{2}\right)\\ \; & +{\left(1-c\right)}^{2}\alpha_{2}^{2v_{1}+2v_{2}}{\left(h^{2}+\alpha_{2}^{2}\right)}^{-v_{1}-d/2}{\left(h^{2}+\alpha_{2}^{2}\right)}^{-v_{2}-d/2}{\left(1\pm 2\beta_{2}\right)}^{2}\\ \; & -c^{2}\alpha_{1}^{2v_{1}+2v_{2}}{\left(h^{2}+\alpha_{1}^{2}\right)}^{-(v_{1}+v_{2})-d}{\left(1\pm 2\beta_{1}\right)}^{2}\\ \; & -2c\left(1-c\right)\alpha_{1}^{v_{1}+v_{2}}\alpha_{2}^{v_{1}+v_{2}}{\left(h^{2}+\alpha_{1}^{2}\right)}^{-(v_{1}+v_{2})/2-d/2}{\left(h^{2}+\alpha_{2}^{2}\right)}^{-(v_{1}+v_{2})/2-d/2}\left(1\pm 2\beta_{1}\right)\left(1\pm 2\beta_{2}\right)\\ \; & -{\left(1-c\right)}^{2}\alpha_{2}^{2v_{1}+2v_{2}}{\left(h^{2}+\alpha_{2}^{2}\right)}^{-(v_{1}+v_{2})-d}{\left(1\pm 2\beta_{2}\right)}^{2}\\ = & \;c\left(1-c\right)\{\alpha_{1}^{2v_{2}}\alpha_{2}^{2v_{1}}{\left(h^{2}+\alpha_{1}^{2}\right)}^{-v_{2}-2/d}{\left(h^{2}+\alpha_{2}^{2}\right)}^{-v_{1}-2/d}\\ & +\alpha_{1}^{2v_{1}}\alpha_{2}^{2v_{2}}{\left(h^{2}+\alpha_{1}^{2}\right)}^{-v_{1}-2/d}{\left(h^{2}+\alpha_{2}^{2}\right)}^{-v_{2}-2/d}\\ & -2\alpha_{1}^{v_{1}+v_{2}}\alpha_{2}^{v_{1}+v_{2}}{\left(h^{2}+\alpha_{2}^{2}\right)}^{-(v_{1}+v_{2})/2-2/d}{\left(h^{2}+\alpha_{2}^{2}\right)}^{-(v_{1}+v_{2})/2-2/d}\}\left(1\pm 2\beta_{1}\right)\left(1\pm 2\beta_{2}\right) \end{array}$$

$$\begin{array}{ccc} = & \;c\left(1-c\right)\{\alpha_{1}^{v_{2}-v_{1}}\alpha_{2}^{v_{1}-v_{2}}{\left(h^{2}+\alpha_{1}^{2}\right)}^{(v_{1}-v_{2})/2}{\left(h^{2}+\alpha_{2}^{2}\right)}^{(v_{2}-v_{1})/2} & \\ & +\alpha_{1}^{v_{1}-v_{2}}\alpha_{2}^{v_{2}-v_{1}}{\left(h^{2}+\alpha_{1}^{2}\right)}^{(v_{2}-v_{1})/2}{\left(h^{2}+\alpha_{2}^{2}\right)}^{(v_{1}-v_{2})/2}-2\}\left(1\pm 2\beta_{1}\right)\left(1\pm 2\beta_{2}\right) & \\ = & \;c\left(1-c\right)\left\{{\left(\frac{\alpha_{1}^{2}\left(h^{2}+\alpha_{2}^{2}\right)}{\alpha_{2}^{2}\left(h^{2}+\alpha_{1}^{2}\right)}\right)}^{(v_{2}-v_{1})/2}+{\left(\frac{\alpha_{2}^{2}\left(h^{2}+\alpha_{1}^{2}\right)}{\alpha_{1}^{2}\left(h^{2}+\alpha_{2}^{2}\right)}\right)}^{(v_{2}-v_{1})/2}-2\right\}\left(1\pm 2\beta_{1}\right)\left(1\pm 2\beta_{2}\right) \end{array}$$

For the necessary part, with $\left(1\pm 2\beta_{1}\right)\left(1\pm 2\beta_{2}\right)\geq 0$, letting h→+∞ in ${\left(\frac{\alpha_{1}^{2}\left(h^{2}+\alpha_{2}^{2}\right)}{\alpha_{2}^{2}\left(h^{2}+\alpha_{1}^{2}\right)}\right)}^{(v_{2}-v_{1})/2}+{\left(\frac{\alpha_{2}^{2}\left(h^{2}+\alpha_{1}^{2}\right)}{\alpha_{1}^{2}\left(h^{2}+\alpha_{2}^{2}\right)}\right)}^{(v_{2}-v_{1})/2}-2$ yields ${\left(\frac{\alpha_{1}^{2}}{\alpha_{2}^{2}}\right)}^{(v_{2}-v_{1})/2}+{\left(\frac{\alpha_{2}^{2}}{\alpha_{1}^{2}}\right)}^{(v_{2}-v_{1})/2}-2$, which is greater than zero, so c∈(0,1) if inequalities (A4) and (A5) hold.
For the sufficient part, other than using Theorem 2 in [22], we can work on ${\left(\frac{\alpha_{1}^{2}\left(h^{2}+\alpha_{2}^{2}\right)}{\alpha_{2}^{2}\left(h^{2}+\alpha_{1}^{2}\right)}\right)}^{(v_{2}-v_{1})/2}$$+{\left(\frac{\alpha_{2}^{2}\left(h^{2}+\alpha_{1}^{2}\right)}{\alpha_{1}^{2}\left(h^{2}+\alpha_{2}^{2}\right)}\right)}^{(v_{2}-v_{1})/2}\geq 2$ by using inequality $a+b\geq \sqrt{2ab}$ along with c∈(0,1), we can prove both inequalities (A4) and (A5). Finally noting that condition c∈(0,1) automatically satisfies inequalities (A7) and (A8), therefore Equation (2) is a valid correlation matrix function if and only if c∈(0,1). □

**Proof of Theorem** **3.** If we let

$$\begin{array}{cc} G_{0}\left(h\right) & =cM\left(h|v,\alpha \right)+\left(1-c\right)M\left(h|v,\alpha^{\prime }\right),\\ G_{1}\left(h\right) & =cM\left(h|v,\alpha \right)\beta_{1}+\left(1-c\right)M\left(h|v,\alpha^{\prime }\right)\beta_{2}, \end{array}$$

in Theorem 1, from which we only need to show $G_{0}\left(h\right)\pm 2G_{1}\left(h\right)$ is a valid covariance matrix function. By Cramér’s theorem in spectral domain, it is to show that the Fourier transform of $G_{0}\left(h\right)\pm 2G_{1}\left(h\right)$ is nonnegative definite. Consider the following Fourier transform matrix function:

$$\begin{array}{c} \left(\begin{array}{cc} f_{11}\left(h\right) & f_{12}\left(h\right)\\ f_{21}\left(h\right) & f_{22}\left(h\right) \end{array}\right), \end{array}$$

where

$$\begin{array}{c} f_{11}\left(h\right)=c\sigma^{2}{\left(h^{2}+\alpha_{1}^{2}\right)}^{-v_{1}-d/2}\cdot \frac{\Gamma \left(v_{1}+d/2\right)\alpha_{1}^{2v_{1}}}{\Gamma \left(v_{1}\right)\pi^{d/2}}\left(1\pm 2\beta_{1}\right)+\\ \left(1-c\right)\sigma^{2}{\left(h^{2}+\alpha_{1}^{\prime 2}\right)}^{-v_{1}-d/2}\cdot \frac{\Gamma \left(v_{1}+d/2\right)\alpha_{1}^{\prime 2v_{1}}}{\Gamma \left(v_{1}\right)\pi^{d/2}}\left(1\pm 2\beta_{2}\right), \end{array}$$

$$\begin{array}{c} f_{12}\left(s\right)=c\rho_{12}\sigma^{2}{\left(||s|\right|}^{2}+\alpha_{12}^{2}{)}^{-v_{12}-d/2}\cdot \frac{\Gamma \left(v_{12}+d/2\right)\alpha_{12}^{2v_{12}}}{\Gamma \left(\left(v_{1}+v_{2}\right)/2\right)\pi^{d/2}}\left(1\pm 2\beta_{1}\right)+\\ \left(1-c\right)\rho_{12}^{\prime }\sigma^{2}{\left(||s|\right|}^{2}+\alpha_{12}^{\prime 2}{)}^{-v_{12}-d/2}\cdot \frac{\Gamma \left(\left(v_{1}+v_{2}\right)/2+d/2\right)\alpha_{12}^{\prime 2v_{12}}}{\Gamma \left(\left(v_{1}+v_{2}\right)/2\right)\pi^{d/2}}\left(1\pm 2\beta_{2}\right), \end{array}$$

$$\begin{array}{c} f_{21}\left(h\right)=f_{12}\left(h\right), \end{array}$$

$$\begin{array}{c} f_{22}\left(h\right)=c\sigma^{2}{\left(h^{2}+\alpha_{2}^{2}\right)}^{-v_{2}-d/2}\cdot \frac{\Gamma \left(v_{2}+d/2\right)\alpha_{2}^{2v_{1}}}{\Gamma \left(v_{2}\right)\pi^{d/2}}\left(1\pm 2\beta_{1}\right)+\\ \left(1-c\right)\sigma^{2}{\left(h^{2}+\alpha_{2}^{\prime 2}\right)}^{-v_{2}-d/2}\cdot \frac{\Gamma \left(v_{2}+d/2\right)\alpha_{2}^{\prime 2v_{1}}}{\Gamma \left(v_{2}\right)\pi^{d/2}}\left(1\pm 2\beta_{2}\right), \end{array}$$

then it is to show that, the condition (6) is equivalent to $f_{11}\left(h\right)f_{22}\left(h\right)-f_{12}\left(h\right)f_{21}\left(h\right)\geq 0$ for any h≥0 based on Cramér’s Theorem, since $f_{11}\left(h\right)\geq 0$, $f_{22}\left(h\right)\geq 0$ have been ensured by condition like (A8) with $\alpha_{1}$ and $\alpha_{2}$ replaced with $\alpha_{i}$ and $\alpha_{i}^{\prime }$ following [13,14], i=1,2. Let $h_{1}=h^{2}+\alpha_{1}^{2},\;h_{1}^{\prime }=h^{2}+\alpha_{1}^{\prime 2},\;h_{2}=h^{2}+\alpha_{2}^{\prime 2},\;h_{2}^{\prime }=h^{2}+\alpha_{2}^{\prime 2},\;c\left(v\right)=\pi^{-d/2}\Gamma \left(v+d/2\right)/\Gamma \left(v\right)$. Note that

(A9) $$\begin{array}{c} f_{11}\left(h\right)f_{22}\left(h\right)-f_{12}\left(h\right)f_{21}\left(h\right)\\ =c^{2}h_{1}^{-v_{1}-\frac{d}{2}}c_{v_{1}}\alpha_{1}^{2v_{1}}\left(1\pm 2\beta_{1}\right)h_{2}^{-v_{2}-\frac{d}{2}}c_{v_{2}}\alpha_{2}^{2v_{2}}\left(1\pm 2\beta_{1}\right)\\ \;+c\left(1-c\right)h_{1}^{-v_{1}-\frac{d}{2}}c_{v_{1}}\alpha_{1}^{2v_{1}}\left(1\pm 2\beta_{1}\right)h_{2}^{\prime -v_{2}-\frac{d}{2}}c_{v_{2}}\alpha_{2}^{\prime 2v_{2}}\left(1\pm 2\beta_{2}\right)\\ \;+c\left(1-c\right)h_{1}^{\prime -v_{1}-\frac{d}{2}}c_{v_{1}}\alpha_{1}^{\prime 2v_{1}}\left(1\pm 2\beta_{2}\right)h_{2}^{-2v_{2}-\frac{d}{2}}c_{v_{2}}\alpha_{2}^{2v_{2}}\left(1\pm 2\beta_{2}\right)\\ \;+{\left(1-c\right)}^{2}h_{1}^{\prime -v_{1}-d/2}c_{v_{1}}\alpha_{1}^{\prime 2v_{1}}\left(1\pm 2\beta_{2}\right)h_{2}^{\prime -v_{2}-d/2}c_{v_{2}}\alpha_{2}^{\prime 2v_{2}}\left(1\pm 2\beta_{2}\right)\\ \;-c^{2}\rho_{12}^{2}h_{12}^{-2v_{12}-d}c_{v_{12}}^{2}\alpha_{12}^{4v_{12}}{\left(1\pm 2\beta_{1}\right)}^{2}\\ \;-2c\left(1-c\right)\rho_{12}\rho_{12}^{\prime }h_{12}^{-v_{12}-\frac{d}{2}}c_{v_{12}}\alpha_{12}^{2v_{12}}\left(1\pm 2\beta_{1}\right)h_{12}^{\prime -v_{12}-\frac{d}{2}}c_{v_{12}}\alpha_{12}^{\prime 2v_{12}}\left(1\pm 2\beta_{2}\right)\\ \;-{\left(1-c\right)}^{2}\rho_{12}^{\prime 2}h_{12}^{\prime -2v_{12}-d}c_{v_{12}}^{2}\alpha_{12}^{\prime 4v_{12}}{\left(1\pm 2\beta_{2}\right)}^{2}\\ =c^{2}{\left(1\pm 2\beta_{1}\right)}^{2}\left(h_{1}^{-v_{1}-\frac{d}{2}}h_{2}^{-v_{2}-\frac{d}{2}}\alpha_{1}^{2v_{1}}\alpha_{2}^{2v_{2}}c_{v_{1}}c_{v_{2}}-\rho_{12}^{2}h_{12}^{-2v_{12}-d}\alpha_{12}^{4v_{12}}c_{v_{12}}^{2}\right)\\ \;+c\left(1-c\right)\left(1\pm 2\beta_{1}\right)\left(1\pm 2\beta_{2}\right)(h_{1}^{-v_{1}-\frac{d}{2}}h_{2}^{\prime -v_{2}-\frac{d}{2}}\alpha_{1}^{2v_{1}}\alpha_{2}^{\prime 2v_{2}}c_{v_{1}}c_{v_{2}}+h_{1}^{\prime -v_{1}-d/2}h_{2}^{-v_{2}-d/2}\alpha_{1}^{\prime 2v_{1}}\alpha_{2}^{2v_{2}}c_{v_{1}}c_{v_{2}}\\ \;-2\rho_{12}\rho_{12}^{\prime }h_{12}^{-v_{12}-\frac{d}{2}}h_{12}^{\prime -v_{12}-\frac{d}{2}}\alpha_{12}^{2v_{12}}\alpha_{12}^{\prime 2v_{12}}c_{v_{12}}^{2})\\ \;+{\left(1-c\right)}^{2}{\left(1\pm 2\beta_{2}\right)}^{2}\left(h_{1}^{\prime -v_{1}-d/2}h_{2}^{\prime -v_{2}-d/2}\alpha_{1}^{\prime 2v_{1}}\alpha_{2}^{\prime 2v_{2}}c_{v_{1}}c_{v_{2}}-\rho_{12}^{\prime 2}h_{12}^{\prime -2v_{12}-d}\alpha_{12}^{\prime 4v_{12}}c_{v_{12}}^{2}\right)\\ =c^{2}{\left(1\pm 2\beta_{1}\right)}^{2}H\left(h\right)+c\left(1-c\right)\left(1\pm 2\beta_{1}\right)\left(1\pm 2\beta_{2}\right)D\left(h\right)+{\left(1-c\right)}^{2}{\left(1\pm 2\beta_{2}\right)}^{2}\tilde{H}\left(h\right). \end{array}$$

Moreover inequalities (8) and (9) imply $H\left(h\right)\geq 0,\tilde{H}\left(h\right)\geq 0$. The proof of this equivalence can be proved if we can show D(h)≥0. Because

$$\begin{array}{c} H\left(h\right)=\frac{\alpha_{1}^{2v_{1}}\alpha_{2}^{2v_{2}}c_{v_{1}}c_{v_{2}}}{{\left(\alpha_{1}^{2}+h^{2}\right)}^{v_{1}+d/2}{\left(\alpha_{2}^{2}+h^{2}\right)}^{v_{2}+d/2}}-\frac{\rho_{12}^{2}\alpha_{12}^{4v_{12}}c_{v_{12}}^{2}}{{\left(\alpha_{12}^{2}+h^{2}\right)}^{2v_{12}+d}}\geq 0, \end{array}$$

$$\begin{array}{c} \tilde{H}\left(h\right)=\frac{\alpha_{1}^{\prime 2v_{1}}\alpha_{2}^{\prime 2v_{2}}c_{v_{1}}c_{v_{2}}}{{\left(\alpha_{1}^{\prime 2}+h^{2}\right)}^{v_{1}+d/2}{\left(\alpha_{2}^{\prime 2}+h^{2}\right)}^{v_{2}+d/2}}-\frac{\rho_{12}^{2}\alpha_{12}^{\prime 4v_{12}}c_{v_{12}}^{2}}{{\left(\alpha_{12}^{\prime 2}+h^{2}\right)}^{2v_{12}+d}}\geq 0, \end{array}$$

by using inequality $a^{2}+b^{2}\geq 2ab$, we have

$$\begin{array}{cc} D(h)= & \frac{\alpha_{1}^{2v_{1}}\alpha_{2}^{\prime 2v_{2}}c_{v_{1}}c_{v_{2}}}{{\left(\alpha_{1}^{2}+h^{2}\right)}^{v_{1}+d/2}{\left(\alpha_{2}^{\prime 2}+h^{2}\right)}^{v_{2}+d/2}}+\frac{\alpha_{1}^{\prime 2v_{1}}\alpha_{2}^{2v_{2}}c_{v_{1}}c_{v_{2}}}{{\left(\alpha_{1}^{\prime 2}+h^{2}\right)}^{v_{1}+d/2}{\left(\alpha_{2}^{2}+h^{2}\right)}^{v_{2}+d/2}}\\ \; & -\frac{2\rho_{12}\rho_{12}^{\prime }\alpha_{12}^{2v_{12}}\alpha_{12}^{\prime 2v_{12}}c_{v_{12}}^{2}}{{\left(\left(\alpha_{12}^{2}+h^{2}\right)\left(\alpha_{12}^{\prime 2}+h^{2}\right)\right)}^{v_{12}+d/2}}\\ \geq & \frac{2c_{v_{1}}c_{v_{2}}\alpha_{1}^{v_{1}}\alpha_{1}^{\prime v_{1}}\alpha_{2}^{v_{2}}\alpha_{2}^{\prime v_{2}}}{{\left(\alpha_{1}^{2}+h^{2}\right)}^{v_{1}/2+d/4}{\left(\alpha_{1}^{\prime 2}+h^{2}\right)}^{v_{1}/2+d/4}{\left(\alpha_{2}^{2}+h^{2}\right)}^{v_{2}/2+d/4}{\left(\alpha_{2}^{\prime 2}+h^{2}\right)}^{v_{2}/2+d/4}}\\ \; & -\frac{2\rho_{12}\rho_{12}^{\prime }\alpha_{12}^{2v_{12}}\alpha_{12}^{\prime 2v_{1}2}c_{v_{12}}^{2}}{{\left(\left(\alpha_{12}^{2}+h^{2}\right)\left(\alpha_{12}^{\prime 2}+h^{2}\right)\right)}^{v_{12}+d/2}}\\ \geq & 0. \end{array}$$

where the last inequality holds since $a^{2}\geq b^{2}$, $a^{\prime 2}\geq b^{\prime 2}$, then $aa^{\prime }\geq bb^{\prime }$ if $a,a^{\prime },b,b^{\prime }$ are all greater than zero. When D(h)=0, it follows from (A9) that $f_{11}\left(h\right)f_{22}\left(h\right)-f_{12}\left(h\right)f_{21}\left(h\right)\geq 0$ holds automatically. So the remaining case is when D(h)>0, and that the RHS of (A9) being greater than or equal to zero for all h≥0 if and only if

$$\begin{array}{c} \underset{h\geq 0,D(h)>0}{inf}\frac{c^{2}{\left(1\pm 2\beta_{1}\right)}^{2}H\left(h\right)+{\left(1-c\right)}^{2}{\left(1\pm 2\beta_{2}\right)}^{2}\tilde{H}\left(h\right)}{\left(1\pm 2\beta_{1}\right)\left(1\pm 2\beta_{2}\right)D\left(h\right)}\geq c\left(c-1\right). \end{array}$$

□

**Proof of Corollary** **1.** The sufficiency can be proved by following the proof of Theorem 2. For the necessary condition, first note that those conditions will ensure (8) and (9) by Theorem 3 in [14] and the inequality (6) will be evaluated in the following cases. (a) When $\alpha_{12}\leq min\left(\alpha_{1},\alpha_{2}\right)$, $\alpha_{12}^{\prime }\leq min\left(\alpha_{1}^{\prime },\alpha_{2}^{\prime }\right)$, $v_{12}=\frac{v_{1}+v_{2}}{2}$, equalities $\rho_{12}^{2}=\frac{c_{v_{1}}c_{v_{2}}}{c_{v_{12}}^{2}}{\left(\frac{\alpha_{12}^{2}}{\alpha_{1}\alpha_{2}}\right)}^{d}$, $\rho_{12}^{\prime 2}=\frac{c_{v_{1}}c_{v_{2}}}{c_{v_{12}}^{2}}{\left(\frac{\alpha_{12}^{\prime 2}}{\alpha_{1}^{\prime }\alpha_{2}^{\prime }}\right)}^{d}$ hold. The minimum zero of LHS of inequality (6) can be reached when h=0, since

$$\begin{array}{cc} H(0) & =\frac{\alpha_{1}^{2v_{1}}\alpha_{2}^{2v_{2}}c_{v_{1}}c_{v_{2}}}{{\left(\alpha_{1}^{2}+0^{2}\right)}^{v_{1}+d/2}{\left(\alpha_{2}^{2}+0^{2}\right)}^{v_{2}+d/2}}-\frac{\rho_{12}^{2}\alpha_{12}^{4v_{12}}c_{v_{12}}^{2}}{{\left(\alpha_{12}^{2}+0^{2}\right)}^{2v_{12}+d}}\\ \; & =\frac{\alpha_{1}^{2v_{1}}\alpha_{2}^{2v_{2}}c_{v_{1}}c_{v_{2}}}{{\left(\alpha_{1}^{2}+0^{2}\right)}^{v_{1}+d/2}{\left(\alpha_{2}^{2}+0^{2}\right)}^{v_{2}+d/2}}-\frac{c_{v_{1}}c_{v_{2}}}{c_{v_{12}}^{2}}{\left(\frac{\alpha_{12}^{\prime 2}}{\alpha_{1}^{\prime }\alpha_{2}^{\prime }}\right)}^{d}\frac{\alpha_{12}^{4v_{12}}c_{v_{12}}^{2}}{{\left(\alpha_{12}^{2}+0^{2}\right)}^{2v_{12}+d}}\\ \; & =\alpha_{1}^{-d}\alpha_{2}^{-d}c_{v_{1}}c_{v_{2}}-\alpha_{1}^{-d}\alpha_{2}^{-d}c_{v_{1}}c_{v_{2}}\\ \; & =0. \end{array}$$

Similarly, $\tilde{H}\left(0\right)=0$. So 0≤c≤1, given that *c* is also constrained by condition like (A8) with $\alpha_{1}$ and $\alpha_{2}$ replaced with $\alpha_{i}$ and $\alpha_{i}^{\prime },i=1,2$. (b) When $\alpha_{12}\geq max\left(\alpha_{1},\alpha_{2}\right)$, $\alpha_{12}^{\prime }\geq max\left(\alpha_{1}^{\prime },\alpha_{2}^{\prime }\right)$,$v_{12}=\frac{v_{1}+v_{2}}{2}$, equalities $\rho_{12}^{2}=\frac{c_{v_{1}}c_{v_{2}}}{c_{v_{12}}^{2}}{\left(\frac{\alpha_{1}}{\alpha_{12}}\right)}^{2v_{1}}{\left(\frac{\alpha_{2}}{\alpha_{12}}\right)}^{2v_{2}}$, $\rho_{12}^{\prime 2}=\frac{c_{v_{1}}c_{v_{2}}}{c_{v_{12}}^{2}}{\left(\frac{\alpha_{1}^{\prime }}{\alpha_{12}^{\prime }}\right)}^{2v_{1}}{\left(\frac{\alpha_{2}^{\prime }}{\alpha_{12}^{\prime }}\right)}^{2v_{2}}$ hold; the minimum zero of LHS of inequality (6) is obtained when h→∞,

$$\begin{array}{cc} H(\infty ) & =\lim_{h\to \infty }\frac{\alpha_{1}^{2v_{1}}\alpha_{2}^{2v_{2}}c_{v_{1}}c_{v_{2}}}{{\left(\alpha_{1}^{2}+h^{2}\right)}^{v_{1}+d/2}{\left(\alpha_{2}^{2}+h^{2}\right)}^{v_{2}+d/2}}-\frac{\rho_{12}^{2}\alpha_{12}^{4v_{12}}c_{v_{12}}^{2}}{{\left(\alpha_{12}^{2}+h^{2}\right)}^{2v_{12}+d}}=0. \end{array}$$

Similarly, $\tilde{H}\left(\infty \right)=0$. So 0≤c≤1, given that *c* is also constrained by condition like (A8) with $\alpha_{1}$ and $\alpha_{2}$ replaced with $\alpha_{i}$ and $\alpha_{i}^{\prime },i=1,2$. □

**Proof of Theorem** **4.** The sufficiency can be established by following the similar proof of Theorem 2. The necessary part will be proved using Cramér’s Theorem for the case p=2 as follows. The Fourier transform of (12) is equal to

$$F\left(h\right)=\left[\begin{array}{cc} c_{\nu_{1}}f_{11}\left(h\right) & c_{\nu_{12}}\rho_{12}f_{12}\left(h\right)\\ c_{\nu_{12}}\rho_{12}f_{21}\left(h\right) & c_{\nu_{2}}f_{22}\left(h\right) \end{array}\right],$$

where $c_{\nu }=\pi^{-d/2}\Gamma \left(\nu +d/2\right)/\Gamma \left(\nu \right)$,

$$\begin{array}{c} f_{ij}\left(h\right)=c{\left(\alpha_{1}\right)}^{v_{i}+v_{j}}{\left(h^{2}+\alpha_{1}^{2}\right)}^{-\frac{v_{i}+v_{j}}{2}-d/2}\beta_{1}^{*}+\left(1-c\right)\alpha_{2}^{v_{i}+v_{j}}{\left(h^{2}+\alpha_{2}^{2}\right)}^{-\frac{v_{i}+v_{j}}{2}-d/2}\beta_{2}^{*}.,i,j=1,2. \end{array}$$

Hence, it is reduced to show that 0≤c≤1 is necessary for F(h) to be nonnegative definite for any h>0 based on Cramér’s Theorem. Which means $f_{11}\left(h\right)\geq 0$, $f_{22}\left(h\right)\geq 0$, and

(A10) $$\begin{array}{cc} c_{v_{1}}c_{v_{2}}f_{11}\left(h\right)f_{22}\left(h\right)-c_{v_{12}}^{2}\rho_{12}^{2}f_{12}\left(h\right)f_{21}\left(h\right) & \geq 0,\;\;h\geq 0 \end{array}$$

From [13], we already know that $f_{11}\left(h\right)\geq 0$ if and only if

(A11) $$\begin{array}{c} {\left\{1-\frac{\alpha_{2}^{d}\left(1-\beta_{1}\right)\left(1+\beta_{2}\right)}{\alpha_{1}^{d}\left(1+\beta_{1}\right)\left(1-\beta_{2}\right)}\right\}}^{-1}\leq c\leq {\left\{1-\frac{\alpha_{1}^{2v_{1}}\left(1+\beta_{1}\right)\left(1-\beta_{2}\right)}{\alpha_{2}^{2v_{1}}\left(1-\beta_{1}\right)\left(1+\beta_{2}\right)}\right\}}^{-1}. \end{array}$$

Also, $f_{22}\left(h\right)\geq 0$ if and only if

(A12) $$\begin{array}{c} {\left\{1-\frac{\alpha_{2}^{d}\left(1-\beta_{1}\right)\left(1+\beta_{2}\right)}{\alpha_{1}^{d}\left(1+\beta_{1}\right)\left(1-\beta_{2}\right)}\right\}}^{-1}\leq c\leq {\left\{1-\frac{\alpha_{1}^{2v_{2}}\left(1+\beta_{1}\right)\left(1-\beta_{2}\right)}{\alpha_{2}^{2v_{2}}\left(1-\beta_{1}\right)\left(1+\beta_{2}\right)}\right\}}^{-1}. \end{array}$$

To evaluate (A10), noting that $c_{v_{1}}c_{v_{2}}=c_{v_{12}}^{2}\rho_{12}^{2}$, We expand the left-hand side of (A10), omitting the positive factor, as follows: Letting $f_{\alpha v}={\left(h^{2}+\alpha^{2}\right)}^{-v-d/2}\alpha^{2v}$,

$$\begin{array}{ccc} \; & c_{v_{1}}c_{v_{2}}\left(c\alpha_{1}^{2v_{1}}{\left(h^{2}+\alpha_{1}^{2}\right)}^{-v_{1}-d/2}\beta_{1}^{*}+\left(1-c\right)\alpha_{2}^{2v_{1}}{\left(h^{2}+\alpha_{2}^{2}\right)}^{-v_{1}-d/2}\beta_{2}^{*}\right)\\ \; & \cdot (c\alpha_{1}^{2v_{2}}{\left(h^{2}+\alpha_{1}^{2}\right)}^{-v_{2}-d/2}\beta_{1}^{*}+\left(1-c\right)\alpha_{2}^{2v_{2}}{\left(h^{2}+\alpha_{2}^{2}\right)}^{-v_{2}-d/2}\beta_{2}^{*})\\ \; & -c_{v_{12}}^{2}\rho_{12}^{2}{\left(c\alpha_{1}^{v_{1}+v_{2}}{\left(h^{2}+\alpha_{1}^{2}\right)}^{-\frac{v_{1}+v_{2}}{2}-d/2}\beta_{1}^{*}+\left(1-c\right)\alpha_{2}^{v_{1}+v_{2}}{\left(h^{2}+\alpha_{2}^{2}\right)}^{-\frac{v_{1}+v_{2}}{2}-d/2}\beta_{2}^{*}\right)}^{2}.\\ = & \;c^{2}f_{\alpha_{1}v_{1}}f_{\alpha_{1}v_{2}}{\left(\beta_{1}^{*}\right)}^{2}+c\left(1-c\right)f_{\alpha_{1}v_{2}}f_{\alpha_{2}v_{1}}\beta_{1}^{*}\beta_{2}^{*}+c\left(1-c\right)f_{\alpha_{1}v_{1}}f_{\alpha_{2}v_{2}}\beta_{1}^{*}\beta_{2}^{*}+{\left(1-c\right)}^{2}f_{\alpha_{2}v_{1}}f_{\alpha_{2}v_{2}}{\left(\beta_{2}^{*}\right)}^{2} & \\ \; & -c^{2}f_{\alpha_{1}v_{12}}f_{\alpha_{1}v_{12}}{\left(\beta_{1}^{*}\right)}^{2}-2c\left(1-c\right)f_{\alpha_{1}v_{12}}f_{\alpha_{2}v_{12}}\beta_{1}^{*}\beta_{2}^{*}-{\left(1-c\right)}^{2}f_{\alpha_{2}v_{12}}f_{\alpha_{2}v_{12}}{\left(\beta_{2}^{*}\right)}^{2} & \\ = & \;c\left(1-c\right)\left\{f_{\alpha_{1}v_{2}}f_{\alpha_{2}v_{1}}+f_{\alpha_{1}v_{1}}f_{\alpha_{2}v_{2}}-2f_{\alpha_{1}v_{12}}f_{\alpha_{2}v_{12}}\right\}\beta_{1}^{*}\beta_{2}^{*} & \\ = & \;c\left(1-c\right)\{\alpha_{1}^{v_{2}-v_{1}}\alpha_{2}^{v_{1}-v_{2}}{\left(h^{2}+\alpha_{1}^{2}\right)}^{(v_{1}-v_{2})/2}{\left(h^{2}+\alpha_{2}^{2}\right)}^{(v_{2}-v_{1})/2} & \\ \; & +\alpha_{1}^{v_{1}-v_{2}}\alpha_{2}^{v_{2}-v_{1}}{\left(h^{2}+\alpha_{1}^{2}\right)}^{(v_{2}-v_{1})/2}{\left(h^{2}+\alpha_{2}^{2}\right)}^{(v_{1}-v_{2})/2}-2\}\beta_{1}^{*}\beta_{2}^{*} & \\ = & \;c\left(1-c\right)\left\{{\left(\frac{\alpha_{1}^{2}\left(h^{2}+\alpha_{2}^{2}\right)}{\alpha_{2}^{2}\left(h^{2}+\alpha_{1}^{2}\right)}\right)}^{(v_{2}-v_{1})/2}+{\left(\frac{\alpha_{2}^{2}\left(h^{2}+\alpha_{1}^{2}\right)}{\alpha_{1}^{2}\left(h^{2}+\alpha_{2}^{2}\right)}\right)}^{(v_{2}-v_{1})/2}-2\right\}\beta_{1}^{*}\beta_{2}^{*} \end{array}$$

With $\beta_{1}^{*}\beta_{2}^{*}\geq 0$, $f_{\alpha_{1}v_{12}}f_{\alpha_{2}v_{12}}\geq 0$, letting h→∞ in ${\left(\frac{\alpha_{1}^{2}\left(h^{2}+\alpha_{2}^{2}\right)}{\alpha_{2}^{2}\left(h^{2}+\alpha_{1}^{2}\right)}\right)}^{(v_{2}-v_{1})/2}+{\left(\frac{\alpha_{2}^{2}\left(h^{2}+\alpha_{1}^{2}\right)}{\alpha_{1}^{2}\left(h^{2}+\alpha_{2}^{2}\right)}\right)}^{(v_{2}-v_{1})/2}-2$ yields ${\left(\frac{\alpha_{1}^{2}}{\alpha_{2}^{2}}\right)}^{(v_{2}-v_{1})/2}+{\left(\frac{\alpha_{2}^{2}}{\alpha_{1}^{2}}\right)}^{(v_{2}-v_{1})/2}-2$, which is greater than zero, so c∈(0,1). Moreover, c∈(0,1) satisfies the constraint in inequality (13). Finally, the nonnegative definiteness of F(h) for any h≥0 implies c∈(0,1). □

**Proof of Theorem** **5.** The proof idea and procedure are similar to those of Theorem 3. Following a similar setup, we consider the Fourier transform of Equation (15) as follows:

$$\left(\begin{array}{cc} f_{11}\left(h\right) & f_{12}\left(h\right)\\ f_{21}\left(h\right) & f_{22}\left(h\right) \end{array}\right)=\left(\begin{array}{cc} cf_{\alpha_{1}v_{1}}\beta_{1}^{*}+\left(1-c\right)f_{\alpha_{1}^{\prime }v_{1}}\beta_{2}^{*} & cf_{\alpha_{12}v_{12}}\beta_{1}^{*}\rho_{12}+\left(1-c\right)f_{\alpha_{12}^{\prime }v_{12}}\beta_{2}^{*}\rho_{12}^{\prime }\\ cf_{\alpha_{12}v_{12}}\beta_{1}^{*}\rho_{12}+\left(1-c\right)f_{\alpha_{12}^{\prime }v_{12}}\beta_{2}^{*}\rho_{12}^{\prime } & cf_{\alpha_{2}v_{2}}\beta_{1}^{*}+\left(1-c\right)f_{\alpha_{2}^{\prime }v_{2}}\beta_{2}^{*} \end{array}\right),$$

where

$$\begin{array}{c} f_{\alpha v}={\left(h^{2}+\alpha^{2}\right)}^{-v-d/2}\cdot \frac{\Gamma \left(v+d/2\right)\alpha^{2v}}{\Gamma \left(v\right)\pi^{d/2}}. \end{array}$$

It remains to show that condition (16) is equivalent to $f_{11}\left(h\right)f_{22}\left(h\right)-f_{12}\left(h\right)f_{21}\left(h\right)\geq 0$ for all h≥0, based on Cramér’s Theorem. Since $f_{11}\left(h\right)\geq 0$ and $f_{22}\left(h\right)\geq 0$ are guaranteed by a condition similar to (A8), with $\alpha_{1}$ and $\alpha_{2}$ replaced by $\alpha_{i}$ and $\alpha_{i}^{\prime }$ respectively, following [13,14] for i=1,2. To this end, note that

(A13) $$\begin{array}{c} f_{11}\left(h\right)f_{22}\left(h\right)-f_{12}\left(h\right)f_{21}\left(h\right)\\ =c^{2}f_{\alpha_{1}v_{1}}f_{\alpha_{2}v_{2}}{\left(\beta_{1}^{*}\right)}^{2}+c\left(1-c\right)f_{\alpha_{1}v_{1}}f_{\alpha_{2}^{\prime }v_{2}}\beta_{1}^{*}\beta_{2}^{*}\\ \;+c\left(1-c\right)f_{\alpha_{1}^{\prime }v_{1}}f_{\alpha_{2}v_{2}}\beta_{1}^{*}\beta_{2}^{*}+{\left(1-c\right)}^{2}f_{\alpha_{1}^{\prime }v_{1}}f_{\alpha_{2}^{\prime }v_{2}}{\left(\beta_{2}^{*}\right)}^{2}\\ \;-c^{2}\rho_{12}^{2}f_{\alpha_{12}v_{12}}^{2}{\left(\beta_{1}^{*}\right)}^{2}-2c\left(1-c\right)\rho_{12}\rho_{12}^{\prime }f_{\alpha_{12}v_{12}}f_{\alpha_{12}^{\prime }v_{12}}\beta_{1}^{*}\beta_{2}^{*}-{\left(1-c\right)}^{2}\rho_{12}^{\prime 2}f_{\alpha_{12}^{\prime }v_{12}}^{2}{\left(\beta_{2}^{*}\right)}^{2}\\ =c^{2}{\left(\beta_{1}^{*}\right)}^{2}\left(f_{\alpha_{1}v_{1}}f_{\alpha_{2}v_{2}}-f_{\alpha_{12}v_{12}}^{2}\rho_{12}^{2}\right)\\ \;+c\left(1-c\right)\beta_{1}^{*}\beta_{2}^{*}\left(f_{\alpha_{1}v_{1}}f_{\alpha_{2}^{\prime }v_{2}}+f_{\alpha_{1}^{\prime }v_{1}}f_{\alpha_{2}v_{2}}-2\rho_{12}\rho_{12}^{\prime }f_{\alpha_{12}v_{12}}f_{\alpha_{12}^{\prime }v_{12}}\right)\\ \;+{\left(1-c\right)}^{2}{\left(\beta_{2}^{*}\right)}^{2}\left(f_{\alpha_{1}^{\prime }v_{1}}f_{\alpha_{2}^{\prime }v_{2}}-f_{\alpha_{12}^{\prime }v_{12}}^{2}\rho_{12}^{2}\right)\\ =c^{2}{\left(\beta_{1}^{*}\right)}^{2}H\left(h\right)+c\left(1-c\right)\beta_{1}^{*}\beta_{2}^{*}D\left(h\right)+{\left(1-c\right)}^{2}{\left(\beta_{2}^{*}\right)}^{2}\tilde{H}\left(h\right). \end{array}$$

Similar to the proof of Theorem 3, inequalities (8) and (9) imply that H(h)≥0, $\tilde{H}\left(h\right)\geq 0$ and D(h)≥0. When D(h)=0, it follows from Equation (A13) that $f_{11}\left(h\right)f_{22}\left(h\right)-f_{12}\left(h\right)f_{21}\left(h\right)\geq 0$ holds naturally. It turns out that D(h)=0 whenever

$$\begin{array}{c} {\left(\frac{1}{\alpha_{1}^{2}+h^{2}}\right)}^{\nu_{1}+d/2}{\left(\frac{1}{\alpha_{2}^{\prime 2}+h^{2}}\right)}^{\nu_{2}+d/2}\alpha_{1}^{2\nu_{1}}\alpha_{2}^{2\nu_{2}}={\left(\frac{1}{\alpha_{1}^{\prime 2}+h^{2}}\right)}^{\nu_{1}+d/2}{\left(\frac{1}{\alpha_{2}^{2}+h^{2}}\right)}^{\nu_{2}+d/2}\alpha_{1}^{\prime 2\nu_{1}}\alpha_{2}^{\prime 2\nu_{2}}. \end{array}$$

Hence the remaining case is when D(h)>0, and the right-hand side of Equation (A13) is nonnegative for all h≥0 if and only if

$$\begin{array}{c} \underset{\begin{array}{c} h\geq 0\\ D(h)>0 \end{array}}{\inf}\frac{c^{2}{\left(\beta_{1}^{*}\right)}^{2}H\left(h\right)+{\left(1-c\right)}^{2}{\left(\beta_{2}^{*}\right)}^{2}\tilde{H}\left(h\right)}{\beta_{1}^{*}\beta_{2}^{*}D\left(h\right)}\geq c\left(c-1\right). \end{array}$$

□
